# Tailorable bimetallic nanozyme mitigates intervertebral disc degeneration by inhibiting oxidative stress and inflammageing

**DOI:** 10.7150/thno.108592

**Published:** 2025-06-09

**Authors:** Anran Zhang, Haiyang Gao, Xianglong Chen, Pengzhi Shi, Zhangrong Cheng, Yuhang Chen, Wang Wu, Wenbo Wu, Cao Yang, Yukun Zhang

**Affiliations:** Department of Orthopedics, Union Hospital, Tongji Medical College, Huazhong University of Science and Technology, Wuhan 430022, China.

**Keywords:** nanozyme, oxidative damage, extracellular matrix, cellular senescence, intervertebral disc degeneration

## Abstract

**Rationale:** Scavenging reactive oxygen species (ROS), modulating extracellular matrix (ECM) anabolism, and preventing senescence of nucleus pulposus cells (NPCs) are crucial factors of treatment approaches for intervertebral disc degeneration (IDD). However, addressing these issues simultaneously has been challenging due to the interactions among the various pathological factors in the disc microenvironment.

**Methods:** Herein, we utilize self-assembly technology and the excellent drug-carrying potential of mesoporous Prussian blue to design a tailorable bimetallic nanozyme platform of a Mn-modified mesoporous Prussian blue loaded with Cibotium barometz (MPB-Mn3-CB) for the treatment of IDD.

**Results:** The enhancement of multiple antioxidant enzyme activities by MPB-Mn3-CB is ascribed to the lower activation energy of the MnN_4_ active site compared to the FeN_4_ active site. *In vitro* and *in vivo* experiments show that MPB-Mn3-CB efficiently scavenges ROS, promotes ECM synthesis, and rescues the senescent phenotype of NPCs by inhibiting the P53 pathway.

**Conclusion:** This work addresses the specific microenvironmental challenges in severe IDD by synchronously tackling multiple interacting pathological factors and provides a potential therapeutic strategy by multifunctional bimetallic nanozyme for IDD treatment.

## Introduction

Low back pain (LBP) poses a significant global health challenge and is a leading cause of dyskinesia. Over the past three decades, the socioeconomic and medical burden of LBP has markedly increased due to population growth and aging 1. Intervertebral disc degeneration (IDD) is a primary contributor to LBP, affecting approximately 40% of individuals worldwide 2. Although the pathophysiology of IDD is multifaceted, there is increasing evidence that oxidative stress, imbalances in extracellular matrix (ECM) anabolism, and cellular senescence are key factors contributing to IDD 3-5. These elements interact synergistically in severe IDD, accelerating disease progression. In the context of endogenous repair strategies aimed at enhancing cell differentiation and preventing cellular senescence, unresolved oxidative stress can lead to ECM degradation by upregulating the production of matrix-degrading enzymes 6. Similarly, in therapeutic approaches targeting inflammation and promoting synthesis of the ECM, senescent nucleus pulposus cells (NPCs) may release inflammatory factors that further disturb the microenvironmental balance of the intervertebral disc (IVD) 7. Given the challenges of effectively mitigating IDD through single or dual therapies, it is imperative to develop a multifunctional repair platform that can simultaneously eliminate excessive oxidative stress, enhance ECM repair, and decelerate cellular senescence.

Cibotium barometz (CB), a widely recognized traditional Chinese medicine, has been extensively employed for millennia in the treatment of various skeletal diseases, including LBP and sciatica 8. Recent studies have confirmed that CB substantially enhances the expression of key anabolic genes in both *in vitro* and *in vivo* models, thereby promoting cartilage repair and ECM synthesis 9, 10. However, the intricate ECM environment in IDD presents a significant challenge for CB to reach the lesion site. Additionally, the poor solubility and short half-life of CB severely limit its effectiveness. Therefore, there is an urgent need for drug delivery systems to improve the stability and efficacy of CB.

Reactive oxygen species (ROS), which mainly include superoxide anion (·O_2_^-^), hydrogen peroxide (H_2_O_2_), and hydroxyl radical (·OH), are a broad category of chemically active molecules or ions that exhibit strong oxidative activity. Under pathological conditions, large amounts of ROS are generated in the closed microenvironment of the IVD. This depolarizes the mitochondrial membrane, leading to an imbalance in the cellular redox state, which causes irreversible cell cycle arrest and ultimately cellular senescence 11. Impaired mitochondrial function in senescent cells simultaneously generates more ROS, creating a vicious circle and accelerating the process of IDD 12. Hence, modulation of ROS levels to protect cells from oxidative stress damage is a critical component of IDD therapy. Nanozymes, a class of nanocatalysts with catalytic activity and remarkable stability similar to natural enzymes, have made significant progress in the field of antioxidants in recent times 13, 14. They remove excess ROS from tissues by imitating the activity of antioxidant enzymes, shielding cells from the damaging impacts of oxidative stress 15, 16. In recent years, research on nanozymes as antioxidants has made significant strides. Among them, Prussian blue (PB) is one of the FDA-approved antidotes. It attracted attention for its good biocompatibility and possession of multiple antioxidant enzyme-like activities, rendering it a potential candidate for the treatment of various ROS-associated diseases 17, 18. Compared to PB, mesoporous Prussian blue (MPB) synthesized by controlled chemical etching possesses a greater specific area, and its hollow structure enhances its ability to deliver drugs effectively 19. However, MPB alone is insufficient to address the complex pathological changes in the IDD microenvironment, necessitating further optimization of its ROS scavenging efficacy and the exploration of its potential mechanisms of action.

Manganese (Mn), as an essential trace element, possesses a remarkable capacity to promote cartilage regeneration and regulate ROS-mediated oxidative stress 20. Mn enhances chondrocyte growth by activating ZIP14, which stimulates integrins to promote osteoblast proliferation and differentiation 21. Additionally, Mn exhibits a protective effect in converting superoxide (·O_2_^-^) to molecular oxygen (O_2_) and hydrogen peroxide (H_2_O_2_) via the enzyme Mn superoxide dismutase (MnSOD), thereby protecting cells from oxidative damage 22. Mn-containing biomaterials are utilized for the treatment of osteoarthritis, atopic dermatitis, and various other therapeutic applications 23, 24. It is important to note that Mn-containing biomaterials are increasingly demonstrating significant potential for mitigating cellular senescence 25.

Herein, our study utilized simple self-assembly to design Mn-modified mesoporous Prussian blue (MPB-Mn). On this basis, the excellent drug-carrying potential of MPB was exploited to load CB into its hollow mesopores, creating a multifunctional nanoplatform for the treatment of IDD. This nanoplatform addresses the specific microenvironmental challenges in severe IDD by synchronously tackling multiple interacting pathological factors. It eliminates excessive oxidative stress while enhancing ECM anabolic processes and effectively mitigating cellular senescence. The density functional theory (DFT) analysis indicated that as the Mn doping increased, the differential charge density also increased. Furthermore, the adsorption and breakdown of ROS showed higher catalytic activity, which confirmed the mechanism of Mn-enhanced nano-antioxidant enzyme activity. The sequencing results demonstrated that the effect of the Mn-modified mesoporous Prussian blue loaded with Cibotium barometz (MPB-Mn3-CB) in delaying cellular senescence occurred via the tumor protein P53 (P53)-related pathway. Meanwhile, both *in vivo* and *in vitro* studies have shown that MPB-Mn3-CB therapy effectively reduced the inflammatory response and enhanced ECM synthesis within the IVD microenvironment. These findings highlight the potential of MPB-Mn3-CB to address key pathological factors in IDD. Our study provides a promising new therapeutic approach for the treatment of IDD.

## Methods

### Preparation of PB and MPB

PB was synthesized using a typical hydrothermal reaction. 198 g of potassium ferricyanide (K_3_[Fe (CN)_6_]) was dissolved with 4.5 g of poly(vinylpyrrolidone) (PVP, K30) in 60 ml of deionized water containing hydrochloric acid (HCL, 0.01 M) and stirred for 30 minutes to obtain a clarified solution. The mixture was then positioned in a muffle furnace and subjected to heating at 80 °C at a rate of 2 °C/minute, maintained for a duration of 20 hours. Subsequently, the product was washed with ethanol and centrifuged three times (10,000 rpm for 10 minutes), and finally, the obtained PB was freeze-dried and stored at 4 °C for further use. Next, PB was converted to MPB using controlled chemical etching. 30 mg of PB and 300 mg of PVP were dissolved in 30 mL of 1 M HCl solution and stirred for 4 hours at room temperature. The container was then placed in a stainless steel autoclave at 140 °C for 4 hours. The MPB was collected after centrifugation (13,000 rpm for 20 minutes) and washed three times with deionized water for further use.

### Preparation of MPB-Mn with different doping levels and MPB-Mn3-CB

The synthesis of MPB-Mn closely resembled that of MPB, with the exception of the addition of Mn. Briefly, 198 g of K_3_[Fe (CN)_6_] was dissolved in 60 mL of deionized water containing HCL (0.01 M) with stirring and 4.5 g of PVP to obtain solution A. Different amounts of MnCl_2_ (0.2, 0.4, and 0.6 mmol) were dissolved in 60 mL of deionized water with magnetic stirring, along with 4.5 g of PVP, to prepare solution B. Solution B was gradually added to solution A, and the reaction was monitored. The resulting mixture was then positioned in a muffle furnace, heated to 80 °C at a rate of 2 °C/minute, and maintained at that temperature for 20 hours. After the reaction, the products were washed and centrifuged with ethanol and deionized water three times (10,000 rpm, 10 minutes), freeze-dried, and then stored at 4 °C. To prepare MPB-Mn3-CB, 1 mg/mL of MPB-Mn3 and 0.2 mg/mL of CB were stirred continuously at room temperature for 12 hours.

### Characterization of morphology and structure

Scanning electron microscopy (SEM) and transmission electron microscopy (TEM) were utilized to assess the morphology of each material group. SEM images were obtained with a Nova field emission microscope (NanoSEM 450). TEM and mapping images were acquired via a Talos F200x TEM microscope (FEI Ltd., USA) operating at 200 kV. X-ray photoelectron spectroscopy (XPS) was employed to analyze the valence states of each element and the incorporation of metal ions into the materials, with all XPS peaks calibrated against the C1s peak at 284.8 eV.

### Drug-loading efficiency (DLE) and encapsulation efficiency (EE) for MPB-Mn3-CB

MPB-Mn3 (5 mg) and CB (5 mg) were mixed in 5 mL of anhydrous ethanol under continuous stirring for 24 hours. The MPB-Mn3-CB composite was isolated via centrifugation, air-dried, and residual CB in the supernatant was quantified using UV-Vis spectrophotometry. Parallel experiments were performed with PB (5 mg) and MPB (5 mg) under identical conditions to evaluate the impact of mesoporous structure on drug-loading efficacy.

DL and EE were calculated using the following equations:

(1) DL (%) = [(M₀ - M₁) / M₂] × 100%

(2) EE (%) = [(M₀ - M₁) / M₀] × 100%

M₀ = Total mass of CB used in formulation; M₁ = Mass of unencapsulated CB in solution; M₂ = Total mass of drug-loaded nanozymes. All experiments were conducted in triplicate (n = 3) to ensure statistical reliability.

### Drug release kinetics analysis

The dynamic dialysis method was employed to evaluate drug release profiles under physiological and acidic microenvironment conditions. Specifically, MPB-Mn3-CB containing 5 mg CB equivalent was suspended in 5.0 mL PBS (containing 0.5% sodium dodecyl sulfate, SDS) under two conditions: Physiological simulation: pH 7.4, 37 °C; Acidic microenvironment: pH 5.3, 37 °C. The dispersion was sealed in dialysis bags (MWCO: 10,000 Da) and immersed in 195 mL PBS receptor medium under continuous agitation (100 rpm). At predetermined intervals, 5 mL aliquots were withdrawn from the release medium for UV-Vis quantification of released CB, with immediate replenishment of 5 mL fresh PBS. Cumulative CB release was calculated to construct drug release profiles. A parallel experiment was conducted using Jelleine-1 hydrogel to encapsulate MPB-Mn3-CB (5 mg CB equivalent), following identical procedures to comparatively assess hydrogel-mediated modulation of drug release kinetics.

### Measurement of SOD-like activity

The superoxide dismutase (SOD) activity of each material group was assessed based on previously published investigations 26, 27. The sample groups, NBT/enzyme working solution, and reaction initiation working solution were combined in a proportion of 20 µL:160 µL:20 µL according to the procedure of Total Superoxide Dismutase Assay Kit with NBT (Beyotime, S0109, China). The mixture was incubated at 37 °C for 30 minutes, and the OD values at 560 nm were measured by a microplate reader.

SOD Inhibition (%): [(Ablank control 1​-Ablank control 2​) - (Asample-Ablank control 3​)​]/ (Ablank control 1​-Ablank control 2​​) × 100%

### Measurement of CAT-like activity

Catalase (CAT) catalyzes the conversion of H_2_O_2_ into H_2_O and O_2_. Using the Catalase Activity Assay Kit (Solarbio, BC0205, China), we assessed the catalase activity of each material group 28. In brief, the principle is that H_2_O_2_ has a characteristic absorption peak at 240 nm, and CAT accelerates the decomposition of H_2_O_2_, resulting in a reduction in absorbance at this wavelength over time. Therefore, the rate of change in absorbance indicates CAT activity.

CAT Activity Change (%): ΔA=A1​-A2​

### Detection of ·O_2_^-^ and •OH by ESR

The ·O_2_^-^ and ·OH scavenging abilities of each material group were analyzed by an electron spin resonance (ESR) spectrometer (Bruker EMX plus spectrometer, Germany). Superoxide anion was produced via the xanthine and xanthine oxidase system, while hydroxyl radicals were produced through the Fenton reaction. Subsequently, they were incubated with each group of nanozymes individually. The produced ·O_2_^-^ and ·OH, along with any residual ·O_2_^-^ and ·OH in the nanozyme, were trapped by 5,5-dimethyl-1-pyrroline-N-oxide (DMPO). The variations in peak intensity among the different groups were detected with the ESR spectrometer.

### DFT calculations methods

Spin-polarized first-principles calculations were performed utilizing density functional theory (DFT) via the Vienna Ab-initio Simulation Package (VASP) 29. The generalized gradient approximation (GGA), specifically the Perdew-Burke-Ernzerhof (PBE) functional, was employed to analyze electronic exchange and correlation effects 30, 31. For Brillouin zone integration during geometrical optimization, uniform G-centered k-point meshes with a resolution of 2π×0.05 Å⁻² and Methfessel-Paxton electronic smearing were applied. All simulations were conducted with a cutoff energy of 500 eV, ensuring total energy convergence within 1 meV per atom. Structural relaxation was carried out until atomic forces fell below 10 meV Å⁻¹ and the stress tensor was within 0.03 GPa of the target value. A vacuum distance of 12 Å was established to guarantee adequate vacuum and prevent interactions between two periods. Van der Waals corrections were integrated into the total Kohn-Sham energy estimates. We employed van der Waals corrections using the DFT-D3 method 32.

### Isolation and culture of human nucleus pulposus cells (NPCs)

To isolate NPCs, fresh and healthy nucleus pulposus (NP) tissue samples were obtained from patients undergoing discectomy. Hank's balanced salt solution was used to preserve the NP tissue specimens during transit, and informed consent was acquired from all patients for tissue sampling. The NP tissues were then cut into small fragments and cleaned twice with PBS to remove any residual surface components. Subsequently, the samples were enzymatically digested using Collagenase II (Biosharp, China) for 4-6 hours at 37 ℃ in a cell incubator. The digested suspension underwent centrifugation at 1200 rpm for five minutes. The precipitate was collected and resuspended in cell culture medium (1:1 DMEM: F12, Gibco, USA) containing 10% fetal bovine serum (FBS, Gibco, USA) and 1% penicillin-streptomycin (Biosharp, China). The medium was first changed after one week and then refreshed every other day until the NPCs reached over 95% confluence and completely covered the bottom of the culture flasks. At this point, the cells were passaged at a ratio of 1:3. NPCs from passages one to three were used for subsequent experiments. The research protocol using patient samples in this experiment was approved by the Ethics Committee of Tongji Medical College, Huazhong University of Science and Technology (No. S341).

### Cell viability assessment

NPCs were inoculated in 96-well plates (NEST, China) at a density of 5000 cells per well and cultured for 24 hours. Following this, cells were treated with varying concentrations from each material group for 1, 3, and 7 days. Subsequently, the cells were incubated with the working solution (100 μL serum free medium and 10 μL CCK8 solution) for 2 hours at 37 °C, utilizing the Cell Counting Kit-8 assay (CCK-8, Beyotime, China). Absorbance was measured at 450 nm to determine the final concentration for each material group. Furthermore, the cells were continuously exposed to these final concentrations under consistent treatment conditions for 1, 3, and 7 days. Cell viability was assessed by measuring absorbance at 450 nm.

The cytotoxicity of each material group was assessed according to the protocol outlined in the Calcein/PI Cell Viability/Cytotoxicity Assay Kit (Beyotime, China). Briefly, NPCs were seeded in 12-well plates at a density of 20,000 cells per well. After removing the residual medium through rinsing with PBS, the Calcein/PI solution was added and incubated for 30 minutes. Subsequently, the cells were examined utilizing a fluorescence microscope (Olympus, BX53). The proportion of Calcein-positive cells was quantified using ImageJ 1.52a (National Institutes of Health, USA).

### Reactive oxygen species (ROS) detection

In order to assess the potential of the materials to scavenge ROS at the cellular level, NPCs were seeded in 12-well plates at a density of 20,000 cells per well. Following a 24-hour co-culture with IL1B (20 ng/ml), each group of NPCs underwent three washes with PBS. The cells were subsequently treated with 2',7'-Dichlorodihydrofluorescein diacetate (DCFH-DA, MedChemExpress, China) for 30 minutes at 37 °C in the dark. Afterwards, observation was conducted using a fluorescence microscope.

### Immunofluorescence analysis

For cellular immunofluorescence, NPCs were seeded onto 18 mm cell culture plates (Biosharp, China) and subjected to various intervention methods as previously described. The cells were fixed in 4% paraformaldehyde for 15 minutes to immobilize them, followed by permeabilization with 0.2% Triton X-100 for 10 minutes at room temperature. Subsequently, the samples were blocked with 1% BSA for 1 hour and incubated with the primary antibody overnight at 4 °C. The following day, the samples were washed 3 times with PBS, each lasting 3 minutes, and then treated with the corresponding fluorescent secondary antibody for 1 hour in the dark. Cell nuclei were stained with DAPI solution (Biosharp, China) for five minutes, after which the slides were examined under a laser scanning confocal microscope (Olympus, BX53) to capture images. Mean fluorescence intensity was quantified by ImageJ 1.52a. The antibodies utilized in this experiment are detailed in Table S4.

### Real-Time quantitative polymerase chain reaction (RT-qPCR)

Total RNA was extracted from NPCs using Trizol reagent (Invitrogen, USA), and the concentration of pure RNA was measured with a NanoDrop spectrophotometer. Following the manufacturer's instructions, a cDNA synthesis kit (Vazyme, China) was employed for reverse transcription and RT-PCR amplification was performed with a real-time PCR system (Life Technologies, Carlsbad, USA). Gene expression levels were normalized to the housekeeping gene GAPDH, and relative expression of each gene was analyzed using the 2 - ΔΔCq method. Primer information used in this experiment is provided in Table S5.

### Western Blot (WB) analysis

Total proteins were extracted from human NPCs using RIPA lysis buffer (Biosharp, China) containing a protease inhibitor (MedChemExpress, China). Protein concentration was determined using the BCA Protein Assay Kit (Beyotime, China). Equal amounts of protein samples from each group were subjected to sodium dodecyl sulphate polyacrylamide gel electrophoresis (SDS-PAGE). The separated proteins were then transferred to PVDF membranes (Millipore, USA). The membranes were blocked using 5% skim milk for 2 hours and subsequently incubated with primary antibody overnight at 4 °C. The following day, the membranes were washed three times with Tris Buffered Saline with Tween 20 (TBST), each wash lasting 10 minutes. After washing, the membranes were treated with the appropriate secondary antibody for 1 hour. Finally, membranes were visualized with ECL Plus (Thermo Fisher Scientific, USA). Membrane gray values from three independent experiments were quantified with ImageJ 1.52a. Details of the antibodies used in this study are provided in Table S4.

### RNA sequencing (RNA-seq) analysis

NPCs underwent RNA extraction with Trizol reagent (Invitrogen, USA) according to standard protocols. Sequencing was subsequently performed on an Illumina Hiseq platform in collaboration with Wuhan SeqHealth Tech Co., Ltd. (project number: KC-P20240103). Briefly, raw data were filtered to remove reads that contain joint, poly-N, and low-quality reads. The filtered data were aligned to the reference genome, and reads aligning to the exon regions of each gene were quantified using featureCounts (Subread-1.5.1; Bioconductor). RPKMs were then calculated. The edgeR package (version 3.12.1) was utilized to screen for differentially expressed genes between groups. Cutoffs of 0.05 for p-value and 2 for fold-change were employed to assess the statistical significance of differences in gene expression. Enrichment analysis of differentially expressed genes was performed using the KOBAS 2.1 software package.

### Senescence-associated β-galactosidase staining

As previously described, we added NPCs to 12-well plates at a density of 20,000 cells per well and exposed them to various interventions. As directed by the SA-β-Gal staining kit (Beyotime, China), the wells were fixed for 15 minutes with the provided staining fixative. Subsequently, the staining working solution was added, and the 12-well plate was sealed with parafilm film and incubated at 37 °C overnight. The following day, senescence-associated expression was observed using fluorescence microscopy and quantified with ImageJ 1.52a.

### RNA interference

Experimental analyses were performed following siRNA transfection. The study utilized: Negative control siRNA (Santa Cruz Biotechnology, sc-37007); MDM2-targeting siRNA (Santa Cruz Biotechnology, sc-29394). Cells were seeded 12 hours prior to transfection, achieving 30-50% confluence by Day 0. Reverse transfection was conducted using 30 nM siRNA complexed with Lipofectamine 2000 (Thermo Fisher Scientific, 11668) in Opti-MEM I Reduced Serum Medium (Gibco, 31985088), following manufacturer protocols. At 24 hours post-transfection, the transfection medium was replaced with complete growth medium prior to subsequent experimental procedures.

### Ubiquitination assay

Cells from experimental groups were lysed using IP lysis buffer (Beyotime Biotechnology, P10013J). Following centrifugation, protein supernatants were collected and denatured by boiling at 100 °C for 10 minutes. For immunoprecipitation, protein A/G magnetic agarose beads (MedChemExpress, HY-K0202) were covalently conjugated with specific primary antibodies through overnight incubation. Ubiquitinated p53 levels were subsequently analyzed by WB using anti-ubiquitin antibodies.

### Establishment of intervertebral disc degeneration (IDD) animal model

The animal experimental protocol was approved by the Animal Ethics Committee of Wuhan Myhalic Biotechnology Co., Ltd (20240304-001). A total of fifteen male Sprague-Dawley rats (8 weeks old, weighing 300 g ± 20 g) were randomly assigned to five groups (n = 3): control group; puncture group; puncture+Gel group; puncture+Gel-MPB-Mn3 group; puncture+Gel-MPB-Mn3-CB group. To induce the IDD model, rats were anesthetized with 0.5 mL/100 g 2% (w/v) pentobarbital (40 mg/kg) via intraperitoneal injection before surgery. The rats were placed in a supine position with their limbs secured. The Co6/7 caudal vertebrae were selected as the modeling site for IDD. Based on the effect of needle size on IDD 33, after sterilizing the caudal puncture site with iodophor, a 21G needle was inserted perpendicular to the tail into the Co6/7 intervertebral disc, rotated 360° to a depth of 5 mm, and withdrawn after a 40-second dwell time to ensure degenerative effects. One week after puncture, each group was treated with slow injections using a 30G needle as follows: control group (no treatment), puncture group (2 μL PBS), gel group (2 μL Gel), puncture+MPB-Mn3 group (2 μL Gel-MPB-Mn3), and puncture+MPB-Mn3-CB group (2 μL Gel-MPB-Mn3-CB). Supplemental injections were given to each group at 4-week intervals as described above. The puncture site was re-sterilized with povidone-iodine, and the rats were housed in cages for regular observation and for changes in feed, bedding, and water.

### Enzyme-linked immunosorbent assay (ELISA)

Serum samples from each rat group were analyzed using ELISA kits for CRP (Beyotime, PC188), IgG (Beyotime, PI482), and IgM (Abcam, ab215085) following manufacturer protocols. Briefly, standards and samples were loaded into antibody-coated microplate wells and incubated to facilitate antigen-antibody binding. After washing, biotinylated detection antibodies were added and incubated. Unbound antibodies were removed by subsequent washes, followed by incubation with HRP-conjugated streptavidin and additional washing. The chromogenic reaction was initiated by adding 3,3',5,5'-tetramethylbenzidine (TMB) substrate (blue coloration), which was terminated to yield a yellow solution. OD at 450 nm was measured after blank subtraction, and protein expression levels were calculated against standard reference curves.

### Radiographic analysis

Following anesthesia via intraperitoneal injection of 0.5 mL/100 g of 2% (w/v) pentobarbital (40 mg/kg), the rats were positioned on an X-ray machine (MULTIX Impact, SIEMENS, Germany) in a supine position for photography and measurement of the disc height index (DHI) using ImageJ 1.52a. IVD coronal T2-weighted magnetic resonance imaging (MRI) images were obtained through MRI scans (3.0T, GE), and the imaging results were evaluated according to the Pfirrmann grading system (Table S6).

### Histological analysis

Rats were euthanized via intraperitoneal injection of an overdose of sodium pentobarbital, and specimens from the caudal vertebrae were collected and fixed in 4% paraformaldehyde. The specimens underwent a gradual decalcification process over three weeks, followed by dehydration and embedding in paraffin. The paraffin blocks were sectioned to a thickness of 4 μm. Sections were stained with hematoxylin-eosin (HE), saffron-O-fast green (Safranin O), and Masson according to standard protocols. Histological status was assessed by appropriate histological grading scales (Table S7, S8).

For tissue immunofluorescence, sections of each group were dewaxed, hydrated, blocked, and incubated overnight at 4 °C with the appropriate primary antibody. The following day, sections were washed with PBS and exposed to the secondary antibody for 1 hour at room temperature in the dark. Subsequently, specimens were observed using a laser-scanning confocal microscope (Olympus BX53) for image acquisition. Mean fluorescence intensity was quantified using ImageJ 1.52a. The antibodies used in this experiment are detailed in Table S4.

### Statistical analysis

Data are presented as means ± SD. Statistical analyses were conducted using GraphPad Prism 8.0 software (La Jolla, CA, USA). Student's t-test was employed to compare two groups, while multiple group comparisons were performed utilizing ANOVA followed by Tukey's post hoc test. A p-value < 0.05 was considered statistically significant.

## Results

### Characterization of MPB and MPB-Mn at various doping levels

**Figure 1:**
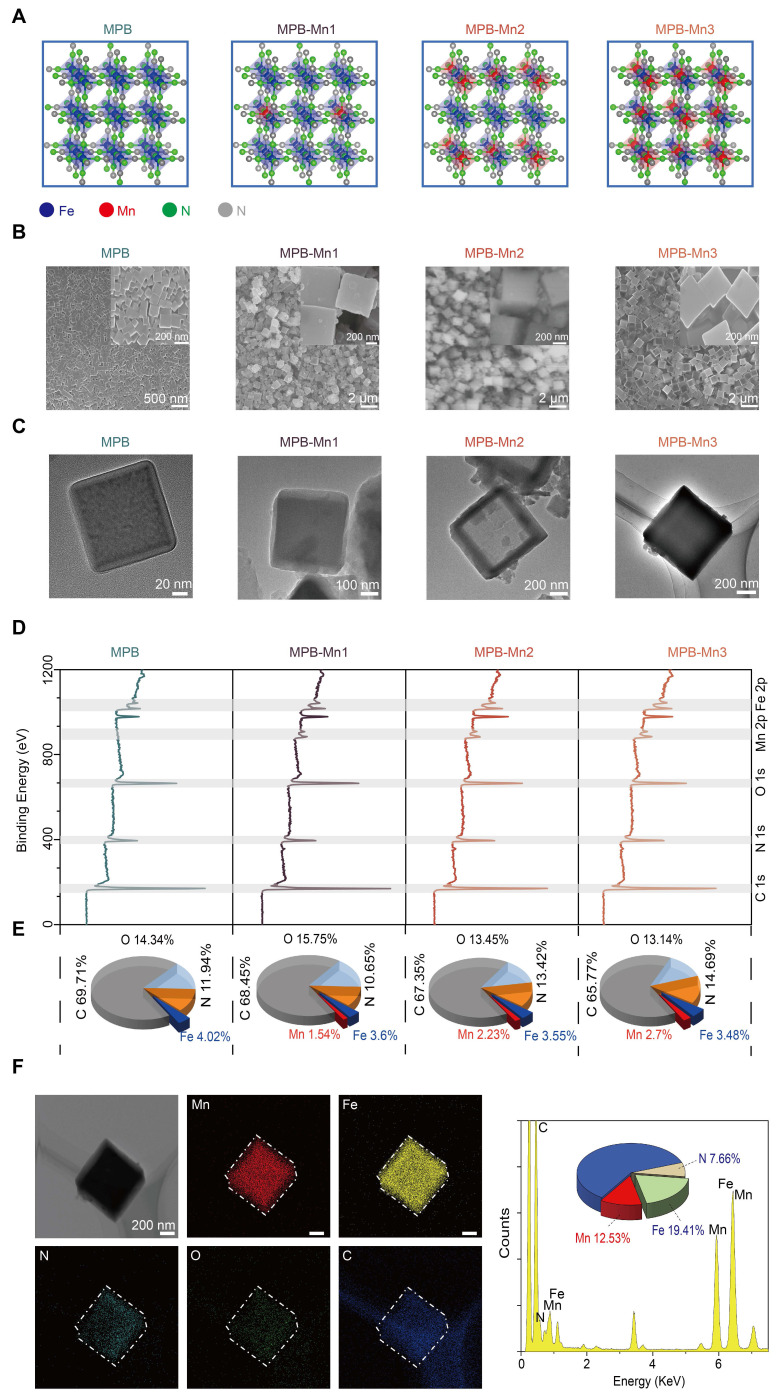
Characterization of MPB and MPB-Mn at various doping levels. (A) Simplified geometrical structure of MPB and MPB-Mn at various doping levels. Fe: blue; Mn: red; N: green; C: gray. (B) Representative SEM images of MPB, MPB-Mn1, MPB-Mn2 and MPB-Mn3. Scale bar (low magnification): 2 µm. Scale bar (high magnification): 200 nm. (C) TEM images of MPB, MPB-Mn1, MPB-Mn2 and MPB-Mn3. Scale bar (low magnification): 200 nm. Scale bar (high magnification): 20 nm. (D) XPS spectra of MPB, MPB-Mn1, MPB-Mn2 and MPB-Mn3. (E) Percentage of each element in MPB, MPB-Mn1, MPB-Mn2 and MPB-Mn3. (F) Elemental mapping images of MPB-Mn3.

By utilizing DFT calculations, the Vienna Ab initio Simulation Package (VASP) computational model explains the geometrical and electronic structure of MPB doped with different Mn ratios to inform the ideal design of MPB-Mn to enhance ROS scavenging. During the experimental synthesis of MPB-Mn, samples were prepared with three distinct Mn doping density gradients, informed by theoretical modeling (Figure 1A). Scanning electron microscopy (SEM) and Transmission electron microscopy (TEM) images show both synthesized MPB and MPB-Mn exhibit a well-dispersed cubic crystalline structure, with dimensions ranging from 100 to 700 nm (Figure 1B-C). The mean dimensions of all three groups of Mn-doped samples are significantly larger than those of MPB, which can be attributed to the substitution of Mn that deforms the host lattice and modifies the interatomic coupling interaction 34. This observation aligns with previously reported crystallographic characteristics of Prussian blue-based nanozymes, confirming that metal doping profoundly influences both lattice architecture and dimensional distribution patterns 35, 36. X-ray photoelectron spectroscopy (XPS) further elucidated the chemical composition and bonding states across samples. As shown in Figure 1D, MPB demonstrated characteristic binding energy peaks at 285.27 eV (C 1s), 397.27 eV (N 1s), 532.27 eV (O 1s), and 708.27 eV (Fe 2p), consistent with established spectral signatures of Prussian blue analogues documented in prior studies 37, 38. The preservation of these fundamental spectral features confirms structural integrity despite Mn incorporation. In comparison, MPB-Mn1 displays peaks at C 1s (285.12 eV), N 1s (397.12 eV), O 1s (532.12 eV), Fe 2p (708.12 eV), and Mn 2p (642.12 eV); MPB-Mn2 shows characteristic peaks at C 1s (285.30 eV), N 1s (397.30 eV), O 1s (532.30 eV), Fe 2p (708.30 eV), and Mn 2p (641.30 eV); and MPB-Mn3 presents characteristic peaks at C 1s (285.35 eV), N 1s (397.35 eV), O 1s (532.35 eV), Fe 2p (708.35 eV), and Mn 2p (642.35 eV). Additionally, the atomic percentages reveal a gradual increase in the Mn content corresponding to the doping density gradient, thereby confirming the successful incorporation of Mn into the MPB (Figure 1E). Furthermore, the elemental mapping demonstrates that the Mn, Fe, C, N, and O elements in MPB-Mn3 are uniformly distributed inside the crystals, thus corroborating the effective synthesis of MPB-Mn (Figure 1F).

### Chemical composition analysis of MPB and MPB-Mn at various doping levels

**Figure 2:**
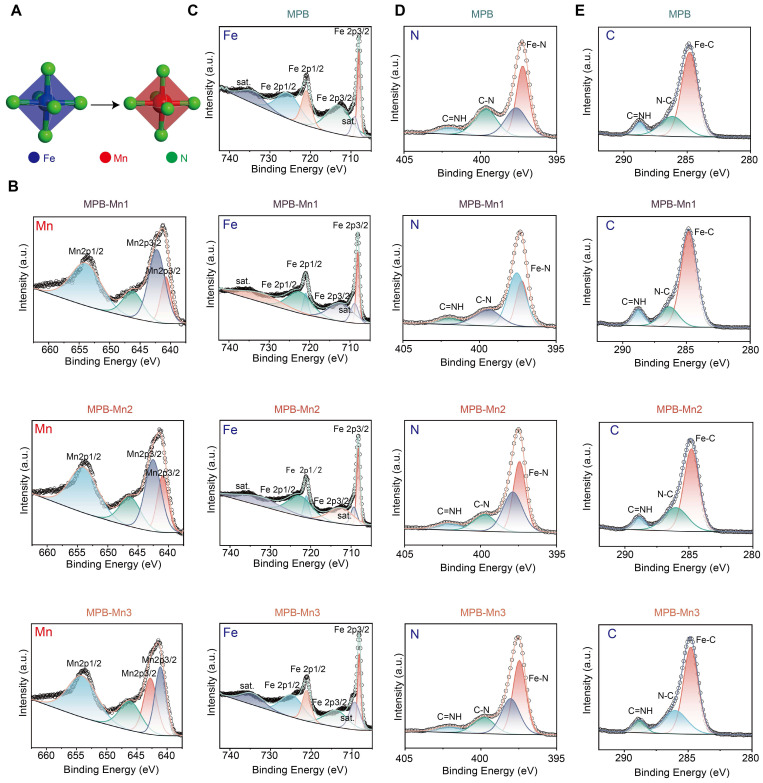
Chemical composition analysis of MPB and MPB-Mn at various doping levels. (A) Schematic diagram of MnN_4_ replacing FeN_4_ in MPB-Mn structure. Fe: blue; Mn: red; N: green. (B) XPS Mn 2p spectra of MPB-Mn1, MPB-Mn2, and MPB-Mn3. (C) XPS Fe 2p spectra of MPB, MPB-Mn1, MPB-Mn2, and MPB-Mn3. (D) XPS N 1s spectra of MPB, MPB-Mn1, MPB-Mn2, and MPB-Mn3. (E) XPS C 1s spectra of MPB, MPB-Mn1, MPB-Mn2, and MPB-Mn3.

To further investigate the chemical composition and elemental valence states of MPB and MPB-Mn with varying doping levels, we conducted high-resolution XPS spectroscopy on each material group. Figure 2A demonstrates the process of Mn substitution at the Fe site of FeN_4_. The Fe 2p spectrum of MPB shows four peaks at 708.02 eV, 713.06 eV, 721.00 eV, and 725.43 eV, which correspond to the Fe2p3/2, Fe2p1/2, with additional peaks representing the associated satellite peaks. This reveals the existence of Fe in MPB in both Fe^2+^ and Fe^3+^ valence states, aligning with the elemental Fe data in MPB. In contrast, Mn was detected in the elemental spectra of MPB-Mn1, MPB-Mn2, and MPB-Mn3 (Figure 2B). The four major peaks of MPB-Mn1 were observed at 640.79 eV, 642.27 eV, 646.19 eV, and 653.87 eV, respectively; the high-resolution XPS Fe2p spectra of MPB-Mn2 were mainly decomposed as 640.97 eV, 642.53 eV, 646.32 eV, and 653.97 eV, while MPB-Mn3 showed peaks at 641.11 eV, 642.78 eV, 646.12 eV, and 654.04 eV. The shifted positions of the Mn2p3/2 and Mn2p1/2 peaks were observed among different groups, which confirmed the variation of the doping amount of Mn among different groups. The change in the valence state of Mn notably resulted in shifts in the positions of the main Fe 2p peaks across different material groups. Specifically, the peaks for MPB-Mn1 were observed at 708.05 eV and 712.29 eV, while those for MPB-Mn2 shifted to 708.20 eV and 712.51 eV. For MPB-Mn3, the main peaks were further observed at 708.22 eV and 713.85 eV. Additionally, the corresponding N 1s peaks exhibited significant shifts as well, indicating that Mn doping alters the coordination environments of both Fe and N (Figure 2C-D). Furthermore, the XPS spectrum of C was divided into three distinct peaks, which were used to perform charge correction for each group. These peaks can be attributed to the Fe-C, N-C=O, and C≡NH functional groups. (Figure 2E). These findings highlight the differences in Mn doping ratios in MPB-Mn1, MPB-Mn2, and MPB-Mn3, further validating the successful synthesis of each material group.

### ROS scavenging capacity of MPB-Mn3 and theoretical studies

**Figure 3:**
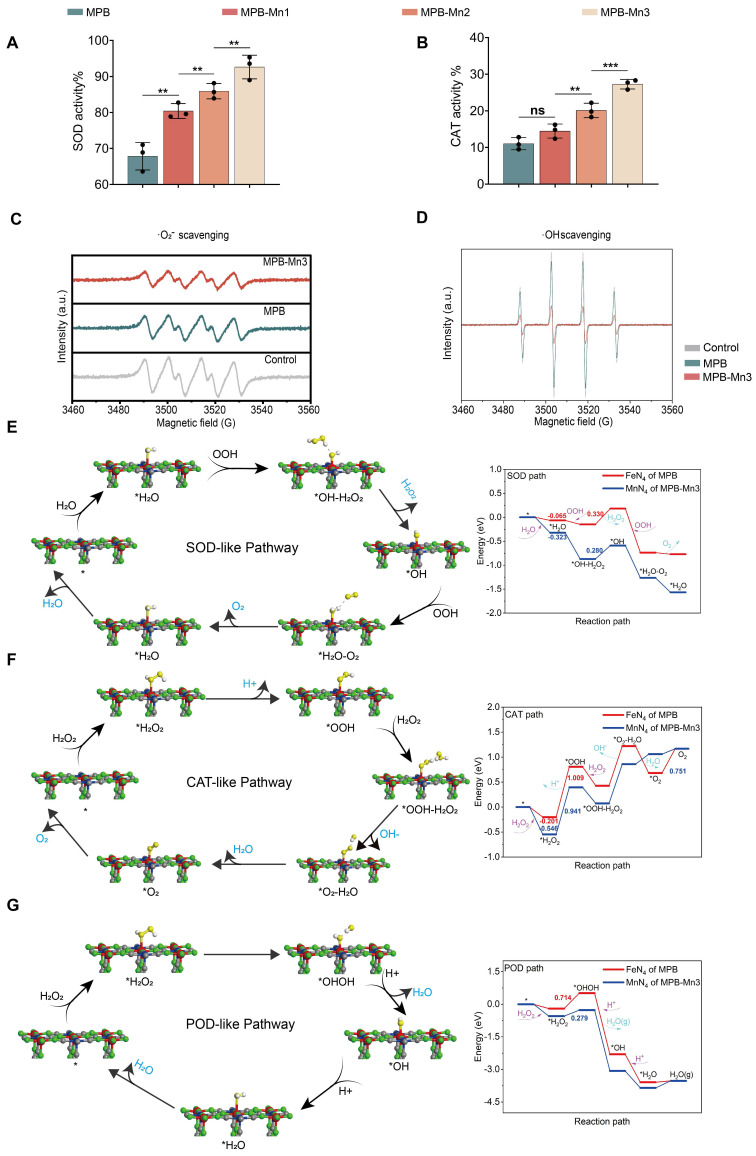
ROS scavenging capacity of MPB-Mn3 and theoretical studies. (A) SOD-like activity of MPB, MPB-Mn1, MPB-Mn2 and MPB-Mn3 (n =3, mean ± SD). (B) CAT-like activity of MPB, MPB-Mn1, MPB-Mn2 and MPB-Mn3 (n =3, mean ± SD). (C) Effects of MPB and MPB-Mn3 on ·O_2_^-^ scavenging detected through ESR spectra. (D) Effects of MPB and MPB-Mn3 on ·OH scavenging detected through ESR spectra. (E) SOD-like pathway of MPB-Mn3 and related Gibbs free energy. (F) CAT-like pathway of MPB-Mn3 and related Gibbs free energy. (G) POD-like pathway of MPB-Mn3 and related Gibbs free energy. (** means P < 0.01 and *** means P < 0.001, ns means no significant difference).

Excessively elevated levels of ROS, such as ·O_2_^-^, ·OH, and H_2_O_2_, can intensify cellular damage and thus aggravate the process of IDD 39, 40. Eliminating the excessively generated ROS to protect cells from oxidative stress damage has become a crucial aspect of IDD treatment. Prior studies have shown that MPB exhibits multiple antioxidant enzyme activities and serves as an excellent ROS scavenger 41, 42. To evaluate the ROS scavenging capacity across different material groups, we first measured Superoxide Dismutase-like (SOD) activity and Catalase-like (CAT) activity. SOD and CAT are typical antioxidant enzymes with distinct physiological functions. SOD catalyzes the removal of electrons from the substrate and transfers them to the hydrogen peroxide reaction, while CAT catalyzes the breakdown of H_2_O_2_ into water and O_2_. The SOD and CAT assay kits demonstrated that MPB, MPB-Mn1, MPB-Mn2, and MPB-Mn3 all possess ROS scavenging capabilities. At the same concentration, MPB-Mn3 showed superior antioxidant effects compared to the other groups, consistent with our anticipated results (Figure 3A-B). To better compare the enzymatic activities of various material groups with conventional nanozymes, we conducted specific activity measurements for CAT, SOD, and Peroxidase (POD) enzymes.

The results demonstrated that all three specific activities of the MPB-Mn series ranked among the top levels of similar antioxidant enzymes, with particularly remarkably high SOD specific activity, proving that compared to other ROS-scavenging nanozymes, MPB-Mn nanozymes exhibit superior elimination capabilities (Figure S1) 43, 44. Further analysis using electron spin resonance (ESR) spectroscopy confirmed the scavenging efficiency of MPB-Mn3 for both ·O_2_^-^ and ·OH. ·O_2_^-^ was generated via the xanthine and xanthine oxidase systems, while hydroxyl radicals resulted from the Fenton reaction. The ESR results indicated a significant reduction in signal intensity for both MPB and MPB-Mn3 compared to the control group, with MPB-Mn3 exhibiting superior scavenging activities (Figure 3C-D). These findings indicate a sequential increase in the ROS scavenging efficiency of MPB, MPB-Mn1, MPB-Mn2, and MPB-Mn3, with MPB-Mn3 exhibiting the highest antioxidant efficacy.

To further elucidate the ROS scavenging mechanisms of MPB-Mn3, we used DFT theoretical calculations to analyze the catalytic pathways and energy profiles of SOD-like, CAT-like, and POD-like. In the SOD-like pathway of MPB-Mn3, H_2_O is adsorbed at the manganese site to form * H_2_O; then OOH grabs a proton of * H_2_O and releases H_2_O_2_ by reduction, which has the highest energy barrier and is the rate-determining step of the entire catalytic reaction process. As illustrated in Figure 3E, applying an electric field to MPB-Mn3 significantly lowers the energy required for the separation of *OH- from H_2_O_2_, facilitating the H_2_O_2_ dissociation. Following that, the resulting *OH intermediate subsequently oxidizes another OOH to O_2_, yielding *H_2_O. Ultimately, after H_2_O dissociation, MPB-Mn3 reverts to its initial state, thereby sustaining the catalytic cycle (Figure S2-S3). For the CAT-like pathway of MPB-Mn3, H_2_O_2_ is initially adsorbed onto the Mn site to generate * H_2_O_2_, which subsequently leads to the formation of the intermediate *OOH via proton loss. This stage is the rate-limiting step for the entire catalytic reaction process. The energy curve indicates a reduction of 0.345 eV in adsorption energy for MPB-Mn3 compared to MPB, as well as a marked decrease in the energy required for subsequent reactions. Thereafter, a second H_2_O_2_ captures the protons of *OOH, resulting in the dissociation of OH- and ultimately yielding H_2_O and O_2_, thereby reverting to the initial state to perpetuate the catalytic cycle (Figure 3F and Figure S4-S5). The POD-like pathway of MPB-Mn3 involves the adsorption of H_2_O_2_ at the Mn active site, followed by the conversion of * H_2_O_2_ to *OHOH, which presents the highest energy barrier and serve as the rate-determining step. The energy profile shows that the necessary reaction energy on MPB-Mn3 is significantly lower than that on MPB. Eventually, two protons are individually absorbed to produce H_2_O, which is then removed and ultimately returned to the initial molecular state (Figure 3G and Figure S6-S7). In summary, DFT demonstrated the excellent ROS scavenging ability of MPB-Mn3 at a theoretical level.

### Structural characterization and mimetic-enzyme capacity of MPB-Mn3-CB

**Figure 4:**
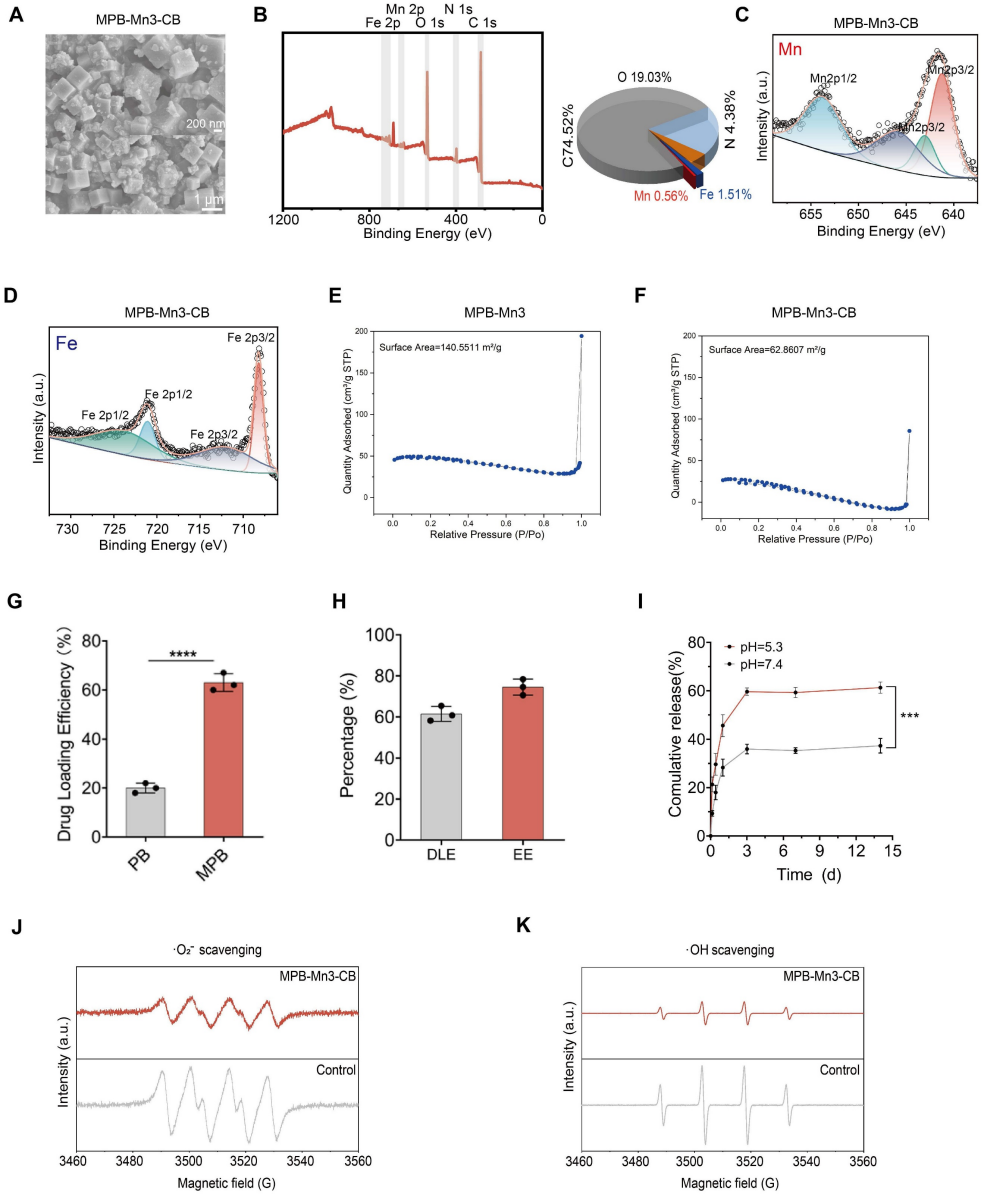
Structural characterization and mimetic-enzyme capacity of MPB-Mn3-CB. (A) Representative SEM images of MPB-Mn3-CB. Scale bar (low magnification): 1 µm. Scale bar (high magnification): 200 nm. (B) XPS spectra and percentage of each element of MPB-Mn3-CB. (C) XPS Mn 2p spectra of MPB-Mn3-CB. (D) XPS Fe 2p spectra of MPB-Mn3-CB. (E, F) The isotherm adsorption-desorption curve of MPB-Mn3 and MPB-Mn3-CB. (G) The drug loading efficiency of PB and MPB (n = 3, means ± SD). (H) DLE and EE evaluation of MPB-Mn3-CB (n = 3, means ± SD). (I) The drug release profiles of MPB-Mn3-CB in PBS at pH=5.3 and 7.4 (n = 3, means ± SD). (J) Effects of MPB-Mn3-CB on ·O_2_^-^ scavenging detected by ESR spectra. (K) Effects of MPB-Mn3-CB on ·OH scavenging detected by ESR spectra. (*** means P < 0.001 and **** means P < 0.0001).

Building on our findings that MPB-Mn3 exhibits superior multiple antioxidant enzyme activities, we proposed to load CB into MPB-Mn3 to facilitate multifunctional repair in IDD. SEM images showed that the surface of MPB-Mn3-CB was uniformly coated with CB, maintaining a crystal structure akin to that of MPB-Mn3, without significant size variation (Figure 4A). The XPS spectra further validated the effective loading of CB (Figure 4B-D). Compared to MPB-Mn3, MPB-Mn3-CB also shows distinct Mn 2p, Fe 2p, C 1s, N 1s, and O 1s peaks, but the peaks of the associated narrow spectra are shifted. The atomic percentages of Mn 2p, Fe 2p, and N 1s were considerably lower than those of MPB-Mn3. To further explore the successful loading of CB in MPB-Mn3, we systematically conducted Brunauer-Emmett-Teller (BET) surface area analyses using nitrogen adsorption-desorption isotherm measurements to evaluate surface property variations before and after drug loading. Experimental results demonstrate that MPB-Mn3-CB remarkably maintains a substantial specific surface area of 62.86 m^2^/g (precisely 62.8607 m^2^/g) post-drug loading (Figure 4E-F). This value aligns closely with those reported for analogous nanocarrier systems in recent literature, confirming the preservation of structural integrity and surface accessibility during the loading process 37. The retained high surface area (>60 m^2^/g) strongly indicates abundant residual active sites available for subsequent therapeutic interactions, which is critical for ensuring optimal drug delivery performance. All the above experimental results indicate that CB was efficiently added into MPB-Mn3. Then We conducted experiments on the effect of the presence or absence of mesopores on drug-loading efficiency. As shown in Figure 4G, comparative studies between PB and MPB carriers reveal a statistically significant enhancement in drug-loading capacity attributable to the hollow mesoporous structure. Then we have systematically evaluated the drug-loading performance of MPB-Mn3-CB to ensure therapeutic efficacy. The results show that the nanozyme exhibits a drug-loading efficiency of 61.2% and encapsulation efficiency of 72.4%, as detailed in Figure 4H. These values surpass conventional drug carriers, demonstrating the structural superiority of our hollow mesoporous architecture for pharmaceutical payload optimization 37, 45. As demonstrated in Figure 4I, the cumulative CB release from the nanozyme system reached 61.33 % over 14 days under simulated cellular microenvironmental conditions. This release profile aligns closely with reported efficiencies of other advanced drug delivery platforms 37, confirming the sustained-release capability of our design. We subsequently evaluated the ROS scavenging capabilities of MPB-Mn3-CB against ·O_2_^-^ and ·OH. ESR results indicated that the loading of CB did not influence the associated ROS scavenging capacity, and MPB-Mn3-CB demonstrated superior antioxidant efficacy (Figure 4J-K). Collectively, these findings affirm the successful synthesis of MPB-Mn3-CB.

### Biocompatibility and ROS scavenging capacity of MPB-Mn3-CB

**Figure 5:**
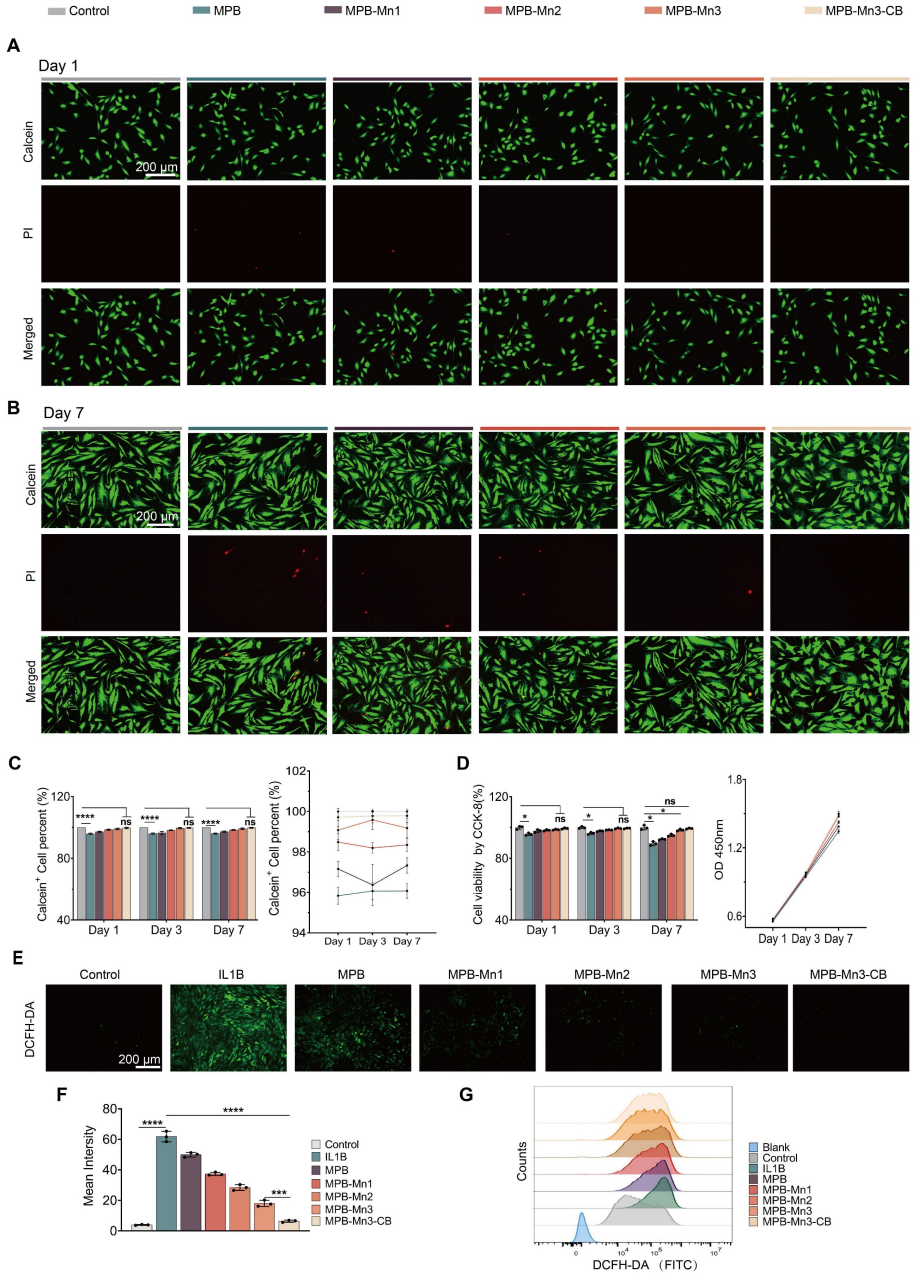
Excellent biocompatibility and ROS scavenging capacity of MPB-Mn3-CB. (A) Representative Live/Dead staining images of the six groups at Day 1. Control group: normal NPCs; MPB group: NPCs treated with MPB; MPB-Mn1 group: NPCs treated with MPB-Mn1; MPB-Mn2 group: NPCs treated with MPB-Mn2; MPB-Mn3 group: NPCs treated with MPB-Mn3; MPB-Mn3-CB group: NPCs treated with MPB-Mn3-CB. Scale bar: 200 μm. (B) Representative Live/Dead staining images of the six groups at Day 7. Control group: normal NPCs; MPB group: NPCs treated with MPB; MPB-Mn1 group: NPCs treated with MPB-Mn1; MPB-Mn2 group: NPCs treated with MPB-Mn2; MPB-Mn3 group: NPCs treated with MPB-Mn3; MPB-Mn3-CB group: NPCs treated with MPB-Mn3-CB. Scale bar: 200 μm. (C) Calcein^+^ cell count obtained from the Live/Dead staining assay and the cell viability profiles of the six groups at different days (n =6, mean ± SD). (D) CCK8 analysis of NPCs and the OD 450nm profiles of the six groups at different days (n =3, mean ± SD). (E) Representative ROS staining images of NPCs by DCFH-DA of the seven groups. Control group: normal NPCs; IL1B group: NPCs treated with IL1B; MPB group: NPCs treated with IL1B and MPB; MPB-Mn1 group: NPCs treated with IL1B and MPB-Mn1; MPB-Mn2 group: NPCs treated with IL1B and MPB-Mn2; MPB-Mn3 group: NPCs treated with IL1B and MPB-Mn3; MPB-Mn3-CB group: NPCs treated with IL1B and MPB-Mn3-CB. Scale bar: 200 μm. (F) Corresponding fluorescence intensity of ROS levels in NPCs under various treatment conditions (n =3, means ± SD). (G) ROS levels in NPCs under various treatment conditions assessed by flow cytometry using DCFH-DA in the FITC channel (530/30 nm bandpass filter) (n =3, means ± SD). (* means P < 0.05 and **** means P < 0.0001, ns means no significant difference).

Given that biocompatibility is essential for the application of nanomaterials in IDD therapy, in order to evaluate the biocompatibility of MPB-Mn3-CB, we first co-cultured with NPCs for 1 and 3 days using different concentrations of each material group. The results of the CCK-8 (Cell Counting Kit-8) assay demonstrated that over 90% of NPCs remained viable at concentrations ranging from 0 to 200 ppm across all material groups within 72 hours (Figure S8). Therefore, we selected 200 ppm as the final concentration for each group in the subsequent cell experiments. The groups at the selected concentrations were co-cultured with NPCs for 1, 3, and 7 days, and their biocompatibility was assessed by the Live/Dead staining kit. The Live/Dead staining images indicated no significant increase in dead cell counts across all groups (Figure 5A-B and Figure S9). Quantitative analysis of Calcein+ cells within the total cell population revealed no statistically significant differences between the MPB-Mn3 and MPB-Mn3-CB groups compared to the control group. Additionally, the time-course analysis of cell viability showed that the viability curve for the MPB-Mn3-CB group closely aligned with that of the control group, while other groups exhibited reduced viability compared to MPB-Mn3-CB (Figure 5C). The CCK-8 assay further corroborated that there were no statistically significant differences in cell counts between the MPB-Mn3-CB and control groups across various time points, while all other groups demonstrated a decreased survival rate relative to the MPB-Mn3-CB group (Figure 5D). Additionally, to enhance quantitative analysis, we conducted corresponding flow cytometry analysis. The results demonstrated that MPB-Mn3 and MPB-Mn3-CB exhibited a significant decrease in PI^+^ cell percent compared to other material groups, and the MPB-Mn3-CB group showed no significant difference compared to the control group. (Figure S10). These findings indicate that MPB-Mn3-CB has excellent biocompatibility. Subsequently, to establish a cellular model of IDD, we proposed treating NPCs with IL1B (interleukin 1 beta) at various doses for 24 hours 46. The appropriate IL1B concentration was determined by detecting the expression of MMP3, IL6, TNF, and COL2A1 by real-time quantitative polymerase chain reaction (RT-qPCR) and western blot (WB). The results indicated that 20 ng/ml of IL1B had the most noticeable effect on the levels of mRNA for MMP3, IL6, TNF, and COL2A1. MMP3, IL6, and TNF were significantly up-regulated, and COL2A1 expression levels were decreased (Figure S11A). The WB results also validated that IL1B at a concentration of 20 ng/ml effectively caused inflammatory degeneration of the intervertebral disc (Figure S11B-C). Thus, the final concentration of IL1B for inducing IDD was selected as 20 ng/ml. To evaluate the potential of MPB-Mn3-CB to remove ROS at the cellular level, NPCs were treated with different material groups at a concentration of 200 ppm for 1 hour, followed by exposure to 20 ng/ml IL1B for 24 hours. The ROS levels were detected using DCFH-DA. According to fluorescence images, ROS levels were significantly higher in the IL1B group than in the control group. The addition of each material group resulted in a drastic decrease in ROS levels (Figure 5E). Quantitative fluorescence analysis demonstrated that the MPB-Mn3-CB group exhibited the greatest ability in eliminating ROS (Figure 5F). Additionally, flow cytometry confirmed that all material groups could reduce ROS levels; however, the MPB-Mn3-CB group exhibited a superior capacity to scavenge ROS compared to the IL1B group, indicating a greater protective effect against oxidative stress (Figure 5G and Figure S12). These results highlight that MPB-Mn3-CB efficiently scavenges excessive ROS while exhibiting excellent biocompatibility, thus protecting NPCs from oxidative stress damage.

### MPB-Mn3-CB restoration of ECM homeostasis in NPCs

During the IDD process, the microenvironment generates numerous inflammatory cytokines, including IL6, TNF, and matrix metalloproteinases (MMP3, MMP13). These cytokines serve to facilitate the breakdown of the ECM, resulting in damage to the IVD 46. To repair IVD, increasing ECM production in NPCs is essential. Collagen II, a key ECM component, is frequently used as a marker to assess ECM function 47. Given that previous experiments demonstrated superior biocompatibility and ROS scavenging ability of the MPB-Mn3 and MPB-Mn3-CB groups compared to other materials, these two groups were selected for subsequent validation experiments. To investigate the role of MPB-Mn3-CB on ECM synthesis, based on the unique microenvironment of intervertebral discs and comparative model evaluations, we adopted an IL1B-induced IDD model to investigate the role of MPB-Mn3-CB in restoring ECM balance (Figure S13) 48-50. Immunofluorescence, RT-qPCR, and WB were used to assess the changes in ECM-related gene and protein expression.

**Figure 6:**
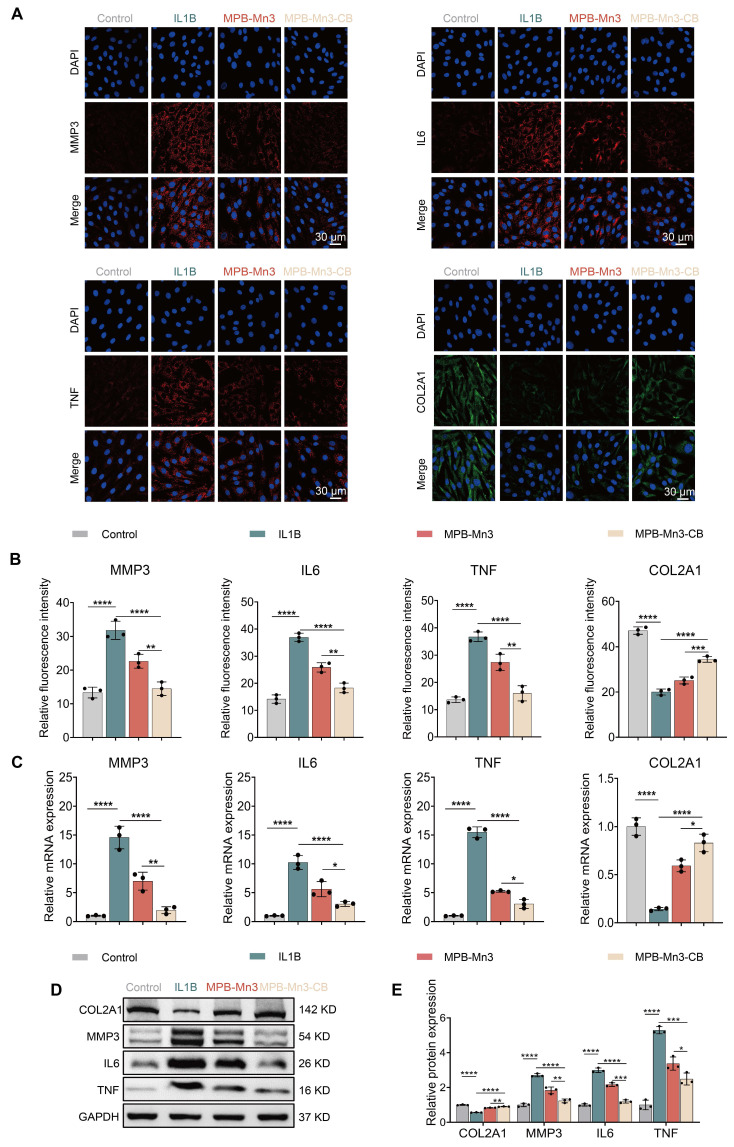
MPB-Mn3-CB promotes ECM anabolism and improves disc microenvironmental homeostasis. (A) Representative immunofluorescence staining images for MMP3, IL6, TNF and COL2A1 of the four groups. Control group: normal NPCs; IL1B group: NPCs treated with IL1B; MPB-Mn3 group: NPCs treated with IL1B and MPB-Mn3; MPB-Mn3-CB group: NPCs treated with IL1B and MPB-Mn3-CB. Scale bar: 30 μm. (B) Quantification of MMP3, IL6, TNF, and COL2A1 fluorescence intensities in NPCs (n = 3, means ± SD). (C) RT-qPCR analysis of MMP3, IL6, TNF, and COL2A1 mRNA expression (n = 3, means ± SD). (D, E) Protein expression levels and quantitative analysis of COL2A1, MMP3, IL6, and TNF in NPCs under various treatment conditions (n = 3, means ± SD). (* means P < 0.05; ** means P < 0.01; *** means P < 0.001 and **** means P < 0.0001).

The results showed that after IL1B intervention, there was a noticeable increase in the fluorescence intensities of MMP3, IL6, and TNF, while the fluorescence intensity of COL2A1 was dramatically reduced in comparison to the control group. These changes indicated that IL1B disrupts the metabolic homeostasis of the ECM (Figure 6A). In contrast, the expression of COL2A1 was significantly elevated in both the MPB-Mn3 and MPB-Mn3-CB groups compared to the IL1B group, and the fluorescence intensities of inflammatory factors and ECM catabolic enzymes were markedly reduced (Figure 6B). These results indicated that MPB-Mn3 and MPB-Mn3-CB effectively promote ECM synthesis in NPCs. Furthermore, RT-qPCR and WB were used to validate these findings. The results were consistent with immunofluorescence data, showing an increase in the expression of MMP3, IL6, and TNF at both the mRNA and protein level in the IL1B group compared to the control group. Conversely, the expression of COL2A1 at both the mRNA and protein levels were found to be decreased (Figure 6C-D). The MPB-Mn3 and MPB-Mn3-CB groups inhibited the expression of MMP3, IL6, and TNF-related proteins, effectively mitigating the negative factors contributing to ECM degradation. Notably, both the MPB-Mn3 and MPB-Mn3-CB groups significantly elevated the mRNA and protein levels of COL2A1, with MPB-Mn3-CB exhibiting the most pronounced restoration of ECM homeostasis across all indices (Figure 6E). These findings demonstrate that MPB-Mn3-CB can effectively counteract the negative effects of IL1B and promote ECM synthesis in the IDD model by regulating the expression levels of the relevant genes and proteins, highlighting their potential to promote IVD repair.

### MPB-Mn3-CB reduces cellular senescence in NPCs via the P53 pathway

**Figure 7:**
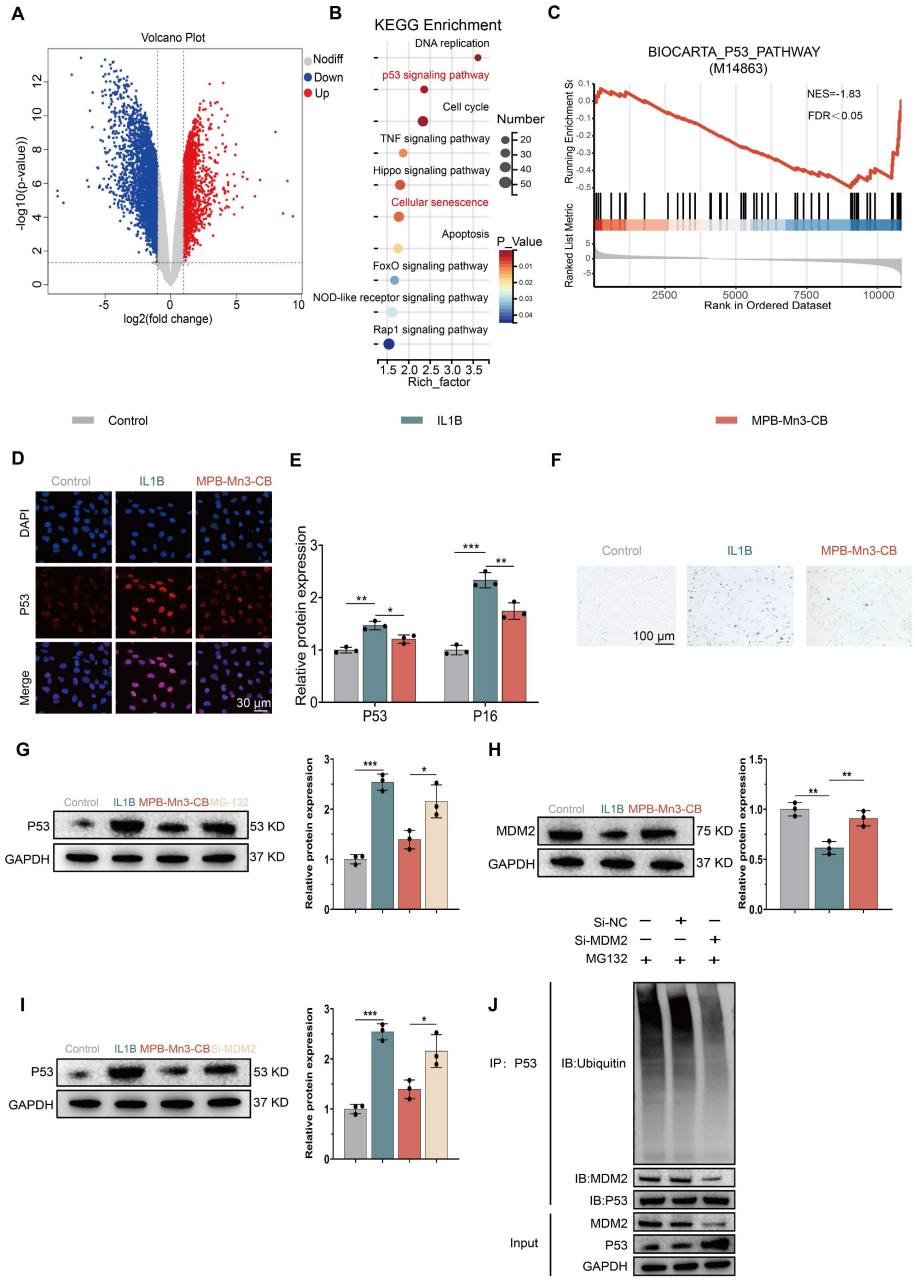
Therapeutic mechanisms of MPB-Mn3-CB on IDD. (A) Volcano plots of the IL1B group treated with MPB-Mn3-CB versus the IL1B group. Red dots represent up-regulated genes, while blue dots represent down-regulated genes. (B) KEGG enrichment pathway analysis of DEGs. (C) GSEA enrichment showing the suppression of the P53 signaling pathway in the MPB-Mn3-CB group. (D) Representative immunofluorescence staining images for P53 of the three groups. Control group: normal NPCs; IL1B group: NPCs treated with IL1B; MPB-Mn3-CB group: NPCs treated with IL1B and MPB-Mn3-CB. Scale bar: 30 μm (n = 3, means ± SD). (E) Quantification of relative protein expression of P53 and P16. (F) Representative SA-β-Gal activity staining images under various treatment conditions. Scale bar: 100 μm. (G) Protein expression levels of P53 in NPCs under various treatment conditions. Control group: normal NPCs; IL1B group: NPCs treated with IL1B; MPB-Mn3-CB group: NPCs treated with IL1B and MPB-Mn3-CB; MG-132 group: NPCs treated with IL1B and MPB-Mn3-CB and MG-132 (n = 3, means ± SD). (H) Protein expression levels of MDM2 in NPCs under various treatment conditions. Control group: normal NPCs; IL1B group: NPCs treated with IL1B; MPB-Mn3-CB group: NPCs treated with IL1B and MPB-Mn3-CB (n = 3, means ± SD). (I) Protein expression levels of P53 in NPCs under various treatment conditions. Control group: normal NPCs; IL1B group: NPCs treated with IL1B; MPB-Mn3-CB group: NPCs treated with IL1B and MPB-Mn3-CB; Si-MDM2 group: NPCs treated with IL1B and MPB-Mn3-CB and Si-MDM2 (n = 3, means ± SD). (J) MDM2 ubiquitinated p53.

To further elucidate the potential mechanism of MPB-Mn3-CB for IDD, RNA-seq was employed to investigate the differentially expressed genes (DEGs) between the MPB-Mn3-CB and the IL1B groups. Compared to the IL1B group, the volcano plots revealed that 1745 genes were up-regulated and 2942 genes were down-regulated in NPCs treated with MPB-Mn3-CB (P < 0.05, fold change >1.5) (Figure 7A). The Kyoto Encyclopedia of Genes and Genomes (KEGG) enrichment analysis revealed that the MPB-Mn3-CB group was significantly enriched in pathways related to the cell cycle, DNA damage response, and cellular senescence (Figure 7B). Furthermore, the Gene Ontology (GO) pathway enrichment analysis also showed that pathways associated with the cell cycle, DNA replication, and mitochondria exhibited significant differences (Figure S14). These findings provide crucial insights into the mechanism by which MPB-Mn3-CB may facilitate IDD repair. Notably, both KEGG and GO analyses significantly enriched the P53-related pathway, a core pathway involved in cellular senescence 51. Through GSEA analysis, we discovered that the MPB-Mn3-CB effectively down-regulates the P53 pathway, indicating its potential to enhance the recovery of IDD by delaying cellular senescence in NPCs (Figure 7C). Building on previous results, we concentrated our validation efforts on the P53 signaling pathway. The immunofluorescence staining results demonstrated a notable increase in the fluorescence intensity of P53 in NPCs treated with IL1B, whereas the corresponding expression of P53 was significantly decreased in MPB-Mn3-CB group, which was consistent with the sequencing results, and proved that p53 is the core molecule in the function of MPB-Mn3-CB (Figure 7D). Subsequently, we systematically measured downstream phenotypic markers associated with the P53 signaling pathway. Quantitative PCR analysis revealed marked alterations in the transcriptional levels of senescence-related molecular markers specifically linked to the P53 pathway, compared with apoptosis- and autophagy-associated molecules. Notably, these P53-mediated senescence-related transcriptional changes were significantly reversed by the senescence inducer Tert-butyl Hydroperoxide (TBHP) (Figure S15). Based on these findings, we further conducted comprehensive validation experiments focusing on the senescence phenotype regulated by the P53 signaling pathway. The WB analysis revealed a substantial increase in the levels of tumor protein P16 (P16) and P53 proteins in NPCs treated with IL1B. However, this effect was somewhat prevented by the administration of MPB-Mn3-CB. The finding was also supported by quantitative analysis of the corresponding indicators (Figure 7E and Figure S16). Then we further verified the above findings by Senescence-Associated β-Galactosidase staining. The stained images demonstrated that MPB-Mn3-CB effectively reduced the occurrence of senescence-positive cells induced by IL1B, suggesting that it enhanced the anti-aging capacity of NPCs (Figure 7F). In summary, these findings indicate that MPB-Mn3-CB delays cellular senescence by inhibiting the P53 signaling pathway in NPCs, contributing to its protective effect against IDD.

To elucidate the molecular mechanism by which MPB-Mn3-CB modulates cellular senescence through the p53 signaling pathway, we systematically investigated the regulatory network of p53 protein. Classical theories posit that p53 downregulation typically results from coordinated transcriptional suppression and post-translational modification-mediated degradation enhancement (e.g., ubiquitin-proteasome system activation) 52-54. qPCR analysis revealed no significant differences in p53 mRNA expression across groups (Figure S17), suggesting post-translational regulation. WB demonstrated that proteasome inhibitor MG-132 treatment substantially reversed MPB-Mn3-CB-induced p53 downregulation (Figure 7G), confirming ubiquitin-proteasome pathway-mediated degradation. Given mouse doubleminute 2 homolog's (MDM2) role as the core E3 ligase regulating p53 ubiquitination 52, we observed significant upregulation of MDM2 at both mRNA and protein levels in MPB-Mn3-CB-treated groups (Figure 7H and Figure S18). siRNA rescue experiments (Figure 7I) showed that MDM2 inhibition counteracted p53 degradation by MPB-Mn3-CB, confirming MDM2's central role in this regulation. Co-immunoprecipitation (IP) assays further validated that MDM2 knockdown significantly reduced p53 ubiquitination levels (Figure 7J). Collectively, these findings demonstrate that MPB-Mn3-CB upregulates MDM2 to enhance p53 ubiquitination, thereby activating the proteasome-dependent degradation pathway and ultimately alleviating the cellular senescence phenotype mediated by p53 hyperactivation.

### MPB-Mn3-CB reverses IDD *in vivo*

#### Radiographic analysis

**Figure 8:**
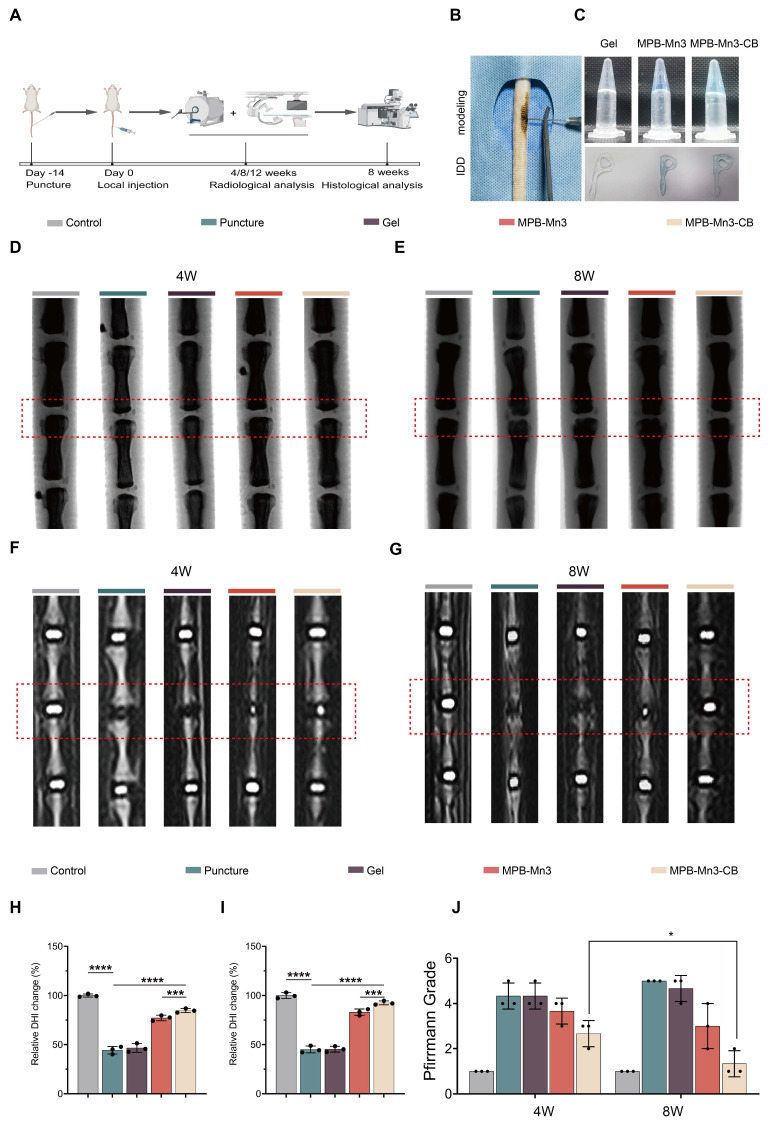
Radiological assessment of MPB-Mn3-CB in *in vivo* experiments. Control group: normal intervertebral disc; Puncture group: punctured intervertebral disc; Gel group: punctured intervertebral disc treated with Gel; MPB-Mn3 group: punctured intervertebral disc treated with Gel-MPB-Mn3 hydrogel; MPB-Mn3-CB group: punctured intervertebral disc treated with Gel-MPB-Mn3-CB hydrogel. (A) Schematic illustration of experimental process that rat models underwent (created with Biorender). (B) The scheme of IDD modeling. (C) The injectable hydrogels and their ability to change shape in different groups. (D, E) Representative X-ray images of caudal vertebra in rats at 4 and 8 weeks. (F, G) Representative MRI images of caudal vertebra in rats at 4 and 8 weeks. (H, I) Quantitative analysis of DHI at 4 and 8 weeks (n = 3, means ± SD). (J) Quantitative of Pfirrmann grades of 4 and 8 weeks (n = 3, means ± SD). (* means P < 0.05; *** means P < 0.001 and **** means P < 0.0001).

To assess the therapeutic impact of MPB-Mn3-CB *in vivo*, we established an IDD model in Sprague-Dawley (SD) rats using needle puncture, as illustrated in Figure 8B. Based on hydrogel's unique advantages as a nanozyme carrier to address IDD microenvironment challenges 55, 56, we selected the Jelleine-1 hydrogel (sequence: PFKLSLH), which exhibits injectability, low immunogenicity, and high biocompatibility. The Jelleine-1 peptide demonstrated a purity of 95.08%, with detailed hydrogel characterization (Figure S19-S21). Comparative release experiments between hydrogel-encapsulated and free MPB-Mn3-CB demonstrate that the hydrogel matrix significantly modulates sustained-release kinetics (Figure S22). This controlled release profile aligns perfectly with the therapeutic requirements for intradiscal material delivery, where prolonged nanoparticle retention is essential to counteract the disc's rapid clearance mechanisms. One week after puncture, the puncture group, Gel group, MPB-Mn3 group, and MPB-Mn3-CB group were injected with PBS, Gel hydrogel, Gel-MPB-Mn3 hydrogel, and Gel-MPB-Mn3-CB hydrogel, respectively. As depicted in Figure S23, MPB-Mn3-CB locally retained in the mouse tail IVD for approximately 28 days. Consequently, we performed monthly local injections of MPB-Mn3-CB to maintain the biological effects. Subsequently, radiographic and histological analyses were performed on the rat caudal spine (Figure 8A). The injectability of the hydrogels is depicted in Figure 8C. Given that imaging findings as critical indicators for IVD healing, we conducted magnetic resonance imaging (MRI) examinations on caudal vertebrae at 4-, 8- and 12-weeks post-intervention. MRI results demonstrated that all treatment groups exhibited significantly superior therapeutic effects compared to the puncture group across different time points (Figure 8F-G and Figure S24A). Notably, the therapeutic outcomes at 8W and 12W showed marked improvements over those at 4W, confirming that the reparative efficacy of MPB-Mn3-CB enhances with prolonged treatment duration (Figure 8J and Figure S25). As no significant difference in therapeutic effect was observed between the 8W and 12W treatment groups, subsequent evaluations primarily focused on the 8W for comparative analysis (Figure S24B). To quantitatively assess the repair effect, we calculated the change in disc height index (DHI) based on radiographic findings (Figure 8D-E). Results revealed the most pronounced disc degeneration in the puncture group, showing striking contrast with the intervertebral height in control group, thereby confirming successful IDD modeling in rat caudal vertebrae. Notably, the Gel group did not show an improvement in IDD. In contrast, both MPB-Mn3 and MPB-Mn3-CB groups demonstrated significant therapeutic effects at 4W and 8W, evidenced by DHI restoration and reduced osteophyte formation (Figure 8H-I). Radiographic analysis confirmed that MPB-Mn3-CB group exhibited the most prominent therapeutic efficacy at both 4W and 8W, effectively reversing IDD-related intervertebral space alterations. MRI, a widely accepted gold standard for diagnosing IDD, was utilized to assess changes in disc water content. The T2-weighted images indicated that healthy IVDs displayed a clear structure with normal signal intensity, while degenerated IVDs exhibited blurred boundaries and lower signal intensity. Imaging results showed significantly decreased signal intensity in the puncture group at 4W and 8W, indicating dramatic loss of IVD hydration. Conversely, both MPB-Mn3 and MPB-Mn3-CB groups exhibited increased signal intensity in IVDs (Figure 8F-G). Pfirrmann grading results at 4W and 8W confirmed significant therapeutic effects in all treatment groups compared to the puncture group (Figure 8H-I). Moreover, the MPB-Mn3-CB group at 8W showed statistically superior outcomes compared to its 4W counterpart, further substantiating that extended treatment duration enhances the reparative efficacy of MPB-Mn3-CB for IDD (Figure 8J).

#### Histological analysis

**Figure 9:**
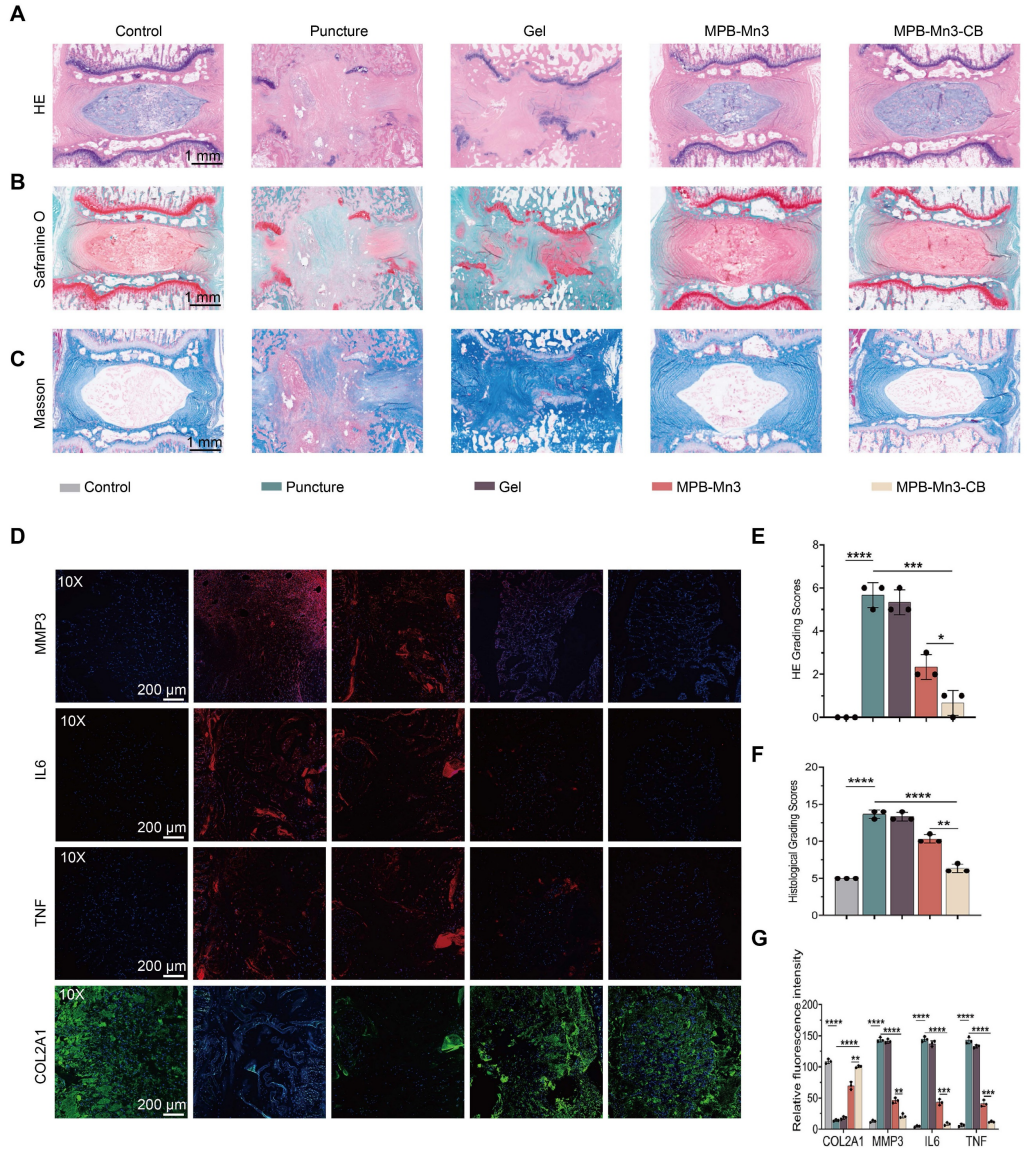
Histological evaluation of MPB-Mn3-CB in animal experiments. (A) Representative H&E staining images of intervertebral discs in Control, Puncture, Gel, MPB-Mn3, and MPB-Mn3-CB groups at 8 weeks. Control group: normal intervertebral disc; Puncture group: punctured intervertebral disc; Gel group: punctured intervertebral disc treated with Gel; MPB-Mn3 group: punctured intervertebral disc treated with MPB-Mn3; MPB-Mn3-CB group: punctured intervertebral disc treated with MPB-Mn3-CB. Scale bar: 1 mm. (B) Representative Safranin-O/Fast Green staining images of different groups at 8 weeks. Scale bar: 1 mm. (C) Representative Masson staining images of different groups at 8 weeks. Scale bar: 1 mm. (D) Representative immunohistochemistry images of MMP3, IL6, TNF, and COL2A1 at 8 weeks. Scale bar: 200 μm. (E) The H&E grading scores of the six groups at 8 weeks (n = 3, means ± SD). (F) The histological grading scores of the six groups at 8 weeks (n = 3, means ± SD). (G) Quantitative of MMP3, IL6, TNF, and Col2A1 immunofluorescence intensity of IVD tissues in different groups (n = 3, means ± SD). (* means P < 0.05, ** means P < 0.01, *** means P < 0.001 and **** means P < 0.0001).

Given the absence of significant differences in therapeutic efficacy between the 8-week and 12-week treatment groups, subsequent investigations primarily focused on analyzing parameter variations across experimental cohorts at the 8-week timepoint (Figure S24B). To investigate the histological differences among the groups, specimens were collected eight weeks post-intervention, followed by complete decalcification and preparation of histological sections. Hematoxylin-eosin (H&E) staining was employed to assess the degree of disc structural alterations initially. Compared to the control group, the intervertebral space in both the Puncture group and the Gel group experienced complete collapse. Additionally, these groups severely disrupted the boundary between the nucleus pulposus (NP) and the annulus fibrosus (AF). In contrast, the MPB-Mn3 and MPB-Mn3-CB groups showed well-defined NP and AF boundaries, with the NP remaining largely intact (Figure 9A). Furthermore, the HE grading scale demonstrated that the MPB-Mn3-CB group had the lowest scores among all groups except the control, significantly lower than those in the Puncture group, thereby suggesting effective facilitation of IVD structural repair by MPB-Mn3-CB (Figure 9E). Additionally, collagen and proteoglycan changes in the IVD were assessed using Safranin O-Fast Green and Masson staining. In comparison to the control group, the Puncture group exhibited a notable infiltration of fibrous tissue, while the Gel group showed no significant difference compared to the Puncture group. Conversely, both MPB-Mn3 and MPB-Mn3-CB groups exhibited no fibrous tissue infiltration or significant damage to the cartilage endplates, indicating a stable proteoglycan and collagen content (Figure 9B-C). Upon quantification of histological scores, it was determined that MPB-Mn3-CB exhibited the most notable enhancement in comparison to the Puncture group (Figure 9F). Overall, these results further affirm the role of MPB-Mn3-CB in improving IDD. Finally, to further verify whether MPB-Mn3-CB enhances ECM formation *in vivo* trials, immunofluorescence analyses were performed to evaluate changes in relevant indicators in the NP tissues. After the puncture procedure, the stained images showed that the fluorescence intensities of MMP3, IL6, and TNF elevated in all treatment groups, while the fluorescence intensities of COL2A1 decreased compared to the control group. The indicators in the MPB-Mn3-CB group were the most similar to those in the control group and showed significant improvements compared to the Puncture group (Figure 9D). The quantitative assessment of fluorescence intensity further supported the conclusion (Figure 9G). Collectively, these findings indicate that MPB-Mn3-CB has a substantial impact on increasing the production of ECM in NP tissues, leading to a marked delay in the progression of IDD. Furthermore, in nanomaterial delivery systems, endocytic internalization serves as the critical pathway for cellular uptake 57. Among various mechanisms, clathrin-mediated endocytosis (CME) and caveolin-mediated endocytosis represent classical cellular internalization routes 58-60. To elucidate the cellular uptake mechanism of MPB-Mn3-CB, we systematically compared the therapeutic effects under pharmacological inhibition of these two pathways using chlorpromazine (MedChemExpress, HY-12708, China) as a clathrin inhibitor and Filipin III (MedChemExpress, HY-N6718, China) as a caveolin inhibitor. Comparative analysis revealed that clathrin pathway inhibition induced significantly greater amelioration of inflammatory markers and extracellular matrix restoration compared to caveolin pathway blockade (Figure S26). This differential response conclusively demonstrates that clathrin-mediated endocytosis predominates in the cellular internalization mechanism of MPB-Mn3-CB nanoparticles. Finally, to evaluate the *in vivo* biocompatibility and toxicology evaluation of MPB-Mn3-CB, we administered it into the NP region of the IVD and harvested the major organs after 8 weeks and 12weeks. Histological examination via HE staining indicated that the major organs from the MPB-Mn3-CB group showed no substantial pathological alterations or increased inflammatory cell infiltration compared to the control group (Figure S27-S28). Furthermore, comprehensive assessments including peripheral blood biochemical analyses, immunogenicity evaluations, and residual Mn ion quantification in intervertebral disc tissues revealed no significant differences between treatment and control groups, collectively demonstrating the MPB-Mn3-CB group's outstanding biocompatibility *in vivo* (Figure S29-S31).

## Discussion and Conclusion

Oxidative stress, imbalance of ECM homeostasis, and NP cellular senescence are all key factors in IDD 61, 62. Firstly, these adverse factors combine to create a detrimental environment for disc cells, ultimately leading to severe pathological changes. Research indicates that dysregulation of ECM anabolism is directly linked to ROS-induced oxidative stress in cells 3; excessively elevated ROS levels and cellular senescence are causally interconnected in the progression of IDD, thereby accelerating the degeneration process 60. Secondly, the compromised IVD microenvironment impairs drug penetration and efficacy at the injury site. Therefore, simultaneously addressing these pathological factors is critical for effective IVD repair.

In recent years, nanozymes have emerged as promising candidates for the therapy of various ROS-related diseases due to their multiple enzyme-like activities and excellent stability 63, 64. PB, an FDA-approved antioxidant exhibiting multiple antioxidant enzyme activities, including SOD, CAT, and POD, is extensively utilized in the treatment of related diseases 65, 66. MPB possesses a greater specific surface area and enhanced drug delivery capacity compared to PB. Considering the multifunctional requirements in IDD therapy and the potential for PB to enhance ROS scavenging, this study utilized MPB and synthesized MPB-Mn3 via a self-assembly procedure with Mn. *In vitro* assays of enzyme-like activity demonstrated that the activity of MPB-Mn3 was markedly superior to MPB in both SOD and CAT. The corresponding DFT analysis further indicated that the differential charge density, together with the adsorption and decomposition activity of ROS in MPB-Mn3, was markedly superior to that of MPB. Moreover, CB, a traditional medicine used for centuries to alleviate LBP, has been shown to markedly enhance anabolic-related gene expression 10. In this study, we loaded CB with the hollow mesoporous structure of MPB to achieve multifunctional repair of IDD. This nanoplatform addresses the specific microenvironmental challenges in severe IDD by synchronously tackling multiple interacting pathological factors.

The experimental results indicate that MPB-Mn3-CB had excellent ability in both biocompatibility and ROS scavenging. In live/dead cell staining assays, the tested materials demonstrated progressively reduced cytotoxicity with increased Mn doping levels and therapeutic agent incorporation. This biocompatibility improvement may be mechanistically attributed to the following aspects: Firstly, iron-based nanomaterials typically exert toxicity partially through Fenton reactions (Fe²⁺ + H₂O₂ → Fe³⁺ + •OH + OH⁻) that generate ROS causing cellular damage 67. Following the substitution of FeN_4_ active sites with MnN_4_ moieties, the nanoparticles likely exhibit diminished free radical generation via Fenton chemistry, thereby manifesting reduced biological toxicity. Secondly, emerging evidence suggests that Mn species may act as electron donors, potentially enhancing M—N bond strength through electron density modulation to suppress demetallization of Fe—N active sites, thereby effectively controlling Fe ion leaching 68. Thirdly, CB, as a conventional anti-inflammatory therapeutic agent, demonstrates potent antioxidant properties that may synergistically mitigate oxidative stress 8. Furthermore, the mesoporous structure of MPB enables controlled release through surface coordination passivation and functional group modifications, which could synergistically regulate metal ion liberation kinetics 69. In the regulation of matrix metabolism, compared to MPB-Mn3, the expression levels of genes related to ECM catabolism, including IL6, TNF, and MMP3, were down-regulated, whereas COL2A1 expression significantly increased, demonstrating its efficacy in restoring ECM homeostasis. Moreover, RNA sequencing results indicated that DNA damage and cellular senescence pathways were significantly enriched in the MPB-Mn3-CB group relative to IL1B-treated NPCs. GSEA analysis demonstrated that the P53 signaling pathway was considerably down-regulated, suggesting the probable mechanism by which MPB-Mn3-CB effectively reversed the senescence phenotype of NPCs. The radiographic and histological scores of the *in vivo* experiments corroborated these findings. Moreover, because the MPB-Mn3-CB nanozyme exhibits CAT -mimetic activity to decompose H₂O₂ into H₂O and O₂. Given the critical role of hypoxia in maintaining IVD homeostasis, we systematically evaluated whether this O_2_ generation might perturb the IVD hypoxic microenvironment (Figure S32). Quantitative PCR analysis of HIF-1α expression—the master regulator of hypoxic adaptation—revealed no statistically significant differences between control and treatment groups, confirming that MPB-Mn3-CB-derived O₂ does not sufficiently activate molecular pathways to disrupt IVD homeostasis. This phenomenon may be mechanistically explained by two factors: (1) The O₂ generation kinetics of Prussian blue nanozymes demonstrate H₂O₂ concentration-dependent characteristics, and despite elevated H₂O₂ levels in degenerated discs, local O₂ production remains constrained by pathological microenvironmental limitations 70; (2) The HIF-1α signaling pathway exhibits intrinsic tolerance to mild O_2_ fluctuations (e.g., 1% → 2% O₂) 71. Nonetheless, our study still has some limitations. First, despite the frequent use of the puncture model in SD rats for IDD induction and treatment, there are still differences between rat and human IVDs. Since rat IVDs fail to accurately replicate the complexity of human IDD due to their lower nutritional requirements 72. Second, because of the limited sample sizes in animal experiments, and given the complexity of human biological systems, the results may not fully reflect the broader population. Moreover, SOD and CAT have conducted cascade antioxidative reactions: ·O_2_^-^ is catalyzed to H₂O₂, and then the accumulated H₂O₂ is converted to H₂O and O₂. In this study, we employed standardized SOD and CAT assay kits to evaluate their respective enzymatic activities, as these kits are widely recognized for their specificity in quantifying individual enzyme activities under controlled conditions 27, 28. However, from a methodological perspective, conventional SOD and CAT assay systems operate independently with distinct substrates and detection mechanisms (nitroblue tetrazolium reduction for SOD and H₂O₂ decomposition kinetics for CAT, making it technically challenging to simultaneously monitor their synergistic interactions in a single reaction system. Therefore, further methodological optimization is required to precisely determine the extent of this cascade reaction. The precise metabolic clearance mechanisms of nanozymes within IVD remain to be fully elucidated. Current studies suggest potential degradation mechanisms for nanozymes in disc microenvironments: 1) lysosomal enzymatic degradation, 2) acidic microenvironment-induced decomposition, and 3) redox reactions during ROS scavenging, ultimately converting them into metal ions 60, 73. Regarding residual metal ions in the local microenvironment, research indicates potential clearance pathways involving local chelation with environmental anions to form insoluble salts, which are subsequently phagocytosed by macrophages and slowly cleared via the lymphatic system 74, 75. Alternatively, another hypothesis proposes that ions may diffuse through interstitial fluid to adjacent vascularized regions (e.g., vertebral endplates), eventually being excreted through renal or hepatobiliary systems 60. However, within the specific context of intervertebral discs, both the clearance mechanisms and optimal research methodologies remain unresolved. Finally, the exact mechanism of MPB-Mn3-CB repair of IDD may require further investigation, including potential effects with other cellular pathways. In conclusion, we have successfully synthesized a novel multifunctional nanozyme, MPB-Mn3-CB, capable of efficiently scavenging excess ROS, promoting ECM anabolism, and inhibiting the senescence of NPCs for IDD repair. Moreover, owing to its superior biocompatibility, it possesses significant promise for clinical translation. We anticipate that it will serve as a promising therapeutic strategy for IDD.

## Supplementary Material

Supplementary figures and tables.

## Figures and Tables

**Scheme 1:**
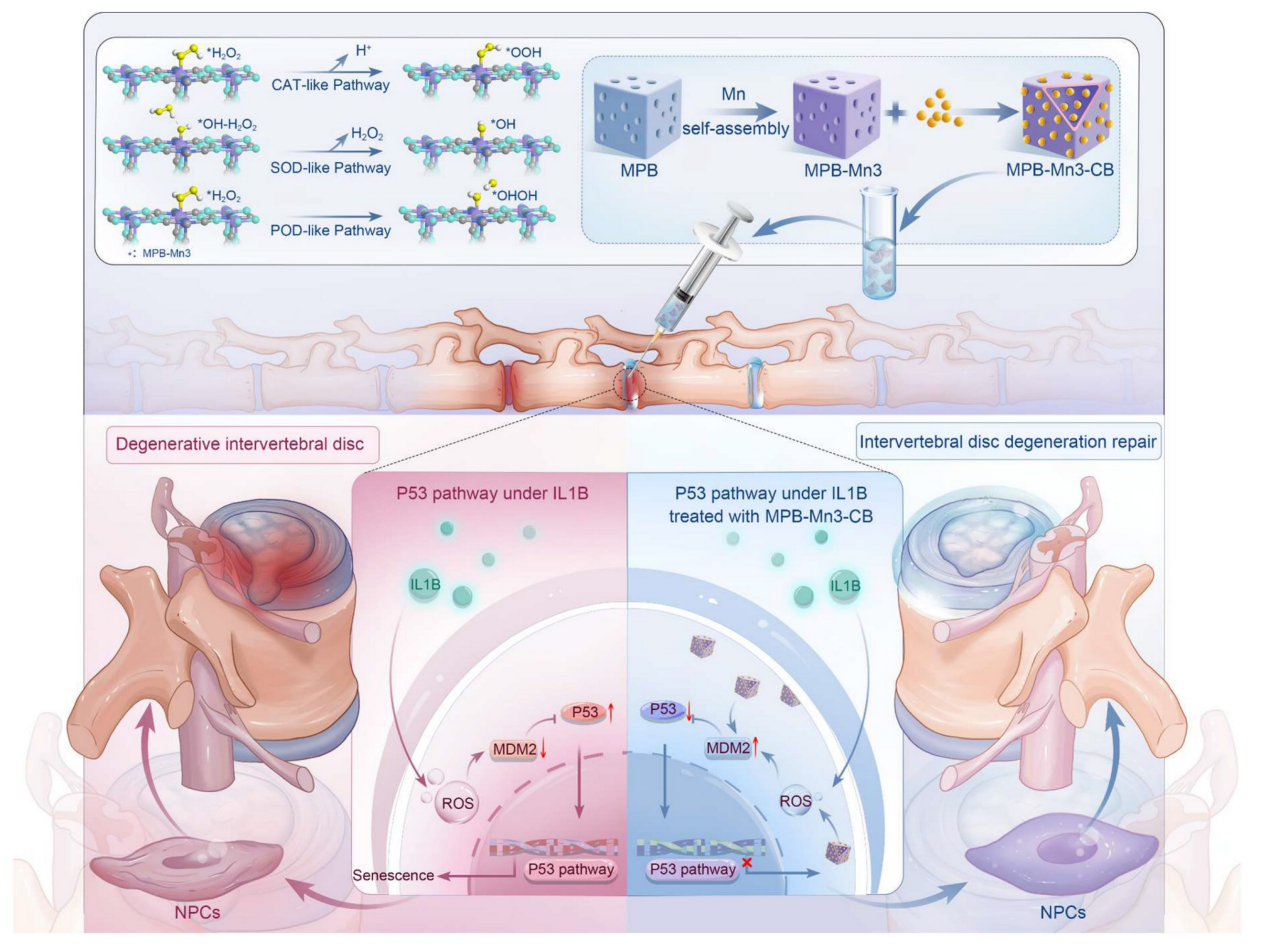
DFT-based bimetallic nanozyme designed for the efficient scavenging of reactive oxygen species, restoring extracellular matrix homeostasis, and inhibiting inflammageing, serve as a multifunctional therapeutic strategy for repairing intervertebral disc degeneration.

## Abbreviations

ROS: reactive oxygen species
ECM: extracellular matrix
NPCs: nucleus pulposus cells
IDD: intervertebral disc degeneration
PB: Prussian blue
MPB: mesoporous Prussian blue
MPB-Mn: Mn-modified mesoporous Prussian blue
CB: Cibotium barometz
MPB-Mn3-CB: Mn-modified mesoporous Prussian blue loaded with Cibotium barometz
LBP: low back pain
IVD: intervertebral disc
·O_2_^-^: superoxide anion
H_2_O_2_: hydrogen peroxide
·OH: hydroxyl radical
Mn: Manganese
O_2_: oxygen
MnSOD: Mn superoxide dismutase
DFT: density functional theory
P53: tumor protein P53
SEM: scanning electron microscopy
TEM: transmission electron microscopy
XPS: X-ray photoelectron spectroscopy
DLE: drug-loading efficiency
EE: encapsulation efficiency
SOD: superoxide dismutase
CAT: Catalase
ESR: electron spin resonance
GGA: generalized gradient approximation
PBE: Perdew-Burke-Ernzerhof
NP: nucleus pulposus
CCK-8: cell counting kit-8 assay
RT-qPCR: real-time quantitative polymerase chain reaction
WB: western blot
RNA-seq: RNA sequencing
ELISA: enzyme-linked immunosorbent assay
DHI: disc height index
MRI: magnetic resonance imaging
HE: hematoxylin-eosin
Safranin O: Safranin O-Fast Green
VASP: Vienna Ab initio Simulation Package
POD: peroxidase
BET: Brunauer-Emmett-Teller
IL1B: interleukin 1 beta
DCFH-DA: 2',7'-dichlorodihydrofluorescein diacetate
DAPI: 4',6-diamidino-2-phenylindole
MMP3: matrix metalloproteinase 3
IL6: interleukin-6
TNF: tumor necrosis factor alpha
COL2A1: collagen type II alpha 1 chain
DEGs: differentially expressed genes
KEGG: kyoto encyclopedia of genes and genomes
GO: gene ontology
TBHP: tert-butyl hydroperoxide
P16: tumor protein P16
MDM2: mouse doubleminute 2 homolog
SD: Sprague-Dawley
AF: annulus fibrosus
CME: clathrin-mediated endocytosis

## References

[1] Hartvigsen J, Hancock MJ, Kongsted A, Louw Q, Ferreira ML, Genevay S (2018). What low back pain is and why we need to pay attention. Lancet.

[2] Cheng Z, Xiang Q, Wang J, Zhang Y (2021). The potential role of melatonin in retarding intervertebral disc ageing and degeneration: A systematic review. Ageing Res Rev.

[3] Chen X, Zhang A, Zhao K, Gao H, Shi P, Chen Y (2024). The role of oxidative stress in intervertebral disc degeneration: Mechanisms and therapeutic implications. Ageing Res Rev.

[4] He X, Hu W, Zhang Y, Chen M, Ding Y, Yang H (2023). Cellular senescence in skeletal disease: mechanisms and treatment. Cell Mol Biol Lett.

[5] Chen S, He T, Zhong Y, Chen M, Yao Q, Chen D (2023). Roles of focal adhesion proteins in skeleton and diseases. Acta Pharm Sin B.

[6] Vo NV, Hartman RA, Yurube T, Jacobs LJ, Sowa GA, Kang JD (2013). Expression and regulation of metalloproteinases and their inhibitors in intervertebral disc aging and degeneration. Spine J.

[7] Zhang Y, Yang B, Wang J, Cheng F, Shi K, Ying L (2020). Cell senescence: A nonnegligible cell state under survival stress in pathology of intervertebral disc degeneration. Oxid Med Cell Longev.

[8] Zhao X, Wu ZX, Zhang Y, Yan YB, He Q, Cao PC (2011). Anti-osteoporosis activity of cibotium barometz extract on ovariectomy-induced bone loss in rats. J Ethnopharmacol.

[9] Huang D, Hou X, Zhang D, Zhang Q, Yan C (2020). Two novel polysaccharides from rhizomes of Cibotium barometz promote bone formation via activating the BMP2/SMAD1 signaling pathway in MC3T3-E1 cells. Carbohydr Polym.

[10] He J, Li X, Wang Z, Bennett S, Chen K, Xiao Z (2019). Therapeutic anabolic and anticatabolic benefits of natural chinese medicines for the treatment of osteoporosis. Front Pharmacol.

[11] Feng C, Yang M, Lan M, Liu C, Zhang Y, Huang B (2017). ROS: Crucial intermediators in the pathogenesis of intervertebral disc degeneration. Oxid Med Cell Longev.

[12] Liu C, Fan L, Guan M, Zheng Q, Jin J, Kang X (2023). A redox homeostasis modulatory hydrogel with GLRX3(+) extracellular vesicles attenuates disc degeneration by suppressing nucleus pulposus cell senescence. ACS Nano.

[13] Xu Y, Luo Y, Weng Z, Xu H, Zhang W, Li Q (2023). Microenvironment-responsive metal-phenolic nanozyme release platform with antibacterial, ROS scavenging, and osteogenesis for periodontitis. ACS Nano.

[14] Yu P, Li Y, Sun H, Zhang H, Kang H, Wang P (2023). Mimicking antioxidases and hyaluronan synthase: A zwitterionic nanozyme for photothermal therapy of osteoarthritis. Adv Mater.

[15] Huang Y, Ren J, Qu X (2019). Nanozymes: classification, catalytic mechanisms, activity regulation, and applications. Chem Rev.

[16] Singh N, Sherin GR, Mugesh G (2023). Antioxidant and prooxidant nanozymes: From cellular redox regulation to next-generation therapeutics. Angew Chem Int Ed Engl.

[17] Zhu D, Wu H, Jiang K, Xu Y, Miao Z, Wang H (2023). Zero-valence selenium-enriched prussian blue nanozymes reconstruct intestinal barrier against inflammatory bowel disease via inhibiting ferroptosis and T cells differentiation. Adv Healthc Mater.

[18] Liu Q, Zhao S, Zhang Y, Fang Q, Liu W, Wu R (2023). Nanozyme-cosmetic contact lenses for ocular surface disease prevention. Adv Mater.

[19] Wu W, Yu L, Pu Y, Yao H, Chen Y, Shi J (2020). Copper-enriched prussian blue nanomedicine for in situ disulfiram toxification and photothermal antitumor amplification. Adv Mater.

[20] Winterbourn CC (2018). Biological production, detection, and fate of hydrogen peroxide. Antioxid Redox Signal.

[21] Sasaki S, Tsukamoto M, Saito M, Hojyo S, Fukada T, Takami M (2018). Disruption of the mouse Slc39a14 gene encoding zinc transporter ZIP14 is associated with decreased bone mass, likely caused by enhanced bone resorption. FEBS Open Bio.

[22] Azadmanesh J, Slobodnik K, Struble LR, Lutz WE, Coates L, Weiss KL (2024). Revealing the atomic and electronic mechanism of human manganese superoxide dismutase product inhibition. Nat Commun.

[23] Wu Y, Zhou Z, Zhang M, Li S, Sun M, Song Z (2023). Hollow manganese dioxide-chitosan hydrogel for the treatment of atopic dermatitis through inflammation-suppression and ROS scavenging. J Nanobiotechnology.

[24] Liu S, Zhang C, Zhou Y, Zhang F, Duan X, Liu Y (2023). MRI-visible mesoporous polydopamine nanoparticles with enhanced antioxidant capacity for osteoarthritis therapy. Biomaterials.

[25] Qu A, Chen Q, Sun M, Xu L, Hao C, Xu C (2024). Sensitive and selective dual-mode responses to reactive oxygen species by chiral manganese dioxide nanoparticles for antiaging skin. Adv Mater.

[26] Liu XW, Zi Y, Liu YE, Zhang YB, Xiang LB, Hou MX (2015). Melatonin exerts protective effect on N2a cells under hypoxia conditions through Zip1/ERK pathway. Neurosci Lett.

[27] Zhang C, Wang H, Yang X, Fu Z, Ji X, Shi Y (2022). Oral zero-valent-molybdenum nanodots for inflammatory bowel disease therapy. Sci Adv.

[28] Gu C, Wang J, Guo M, Sui M, Lu H, Liu G (2018). Extracellular degradation of tetrabromobisphenol a via biogenic reactive oxygen species by a marine pseudoalteromonas sp. Water Res.

[29] Kresse G, Furthmüller J (1996). Efficiency of ab-initio total energy calculations for metals and semiconductors using a plane-wave basis set. Comput Mater Sci.

[30] Perdew JP, Burke K, Ernzerhof M (1996). Generalized gradient approximation made simple. Phys Rev Lett.

[31] Blöchl PE (1994). Projector augmented-wave method. Phys Rev B Condens Matter.

[32] Grimme S, Antony J, Ehrlich S, Krieg H (2010). A consistent and accurate ab initio parametrization of density functional dispersion correction (DFT-D) for the 94 elements H-Pu. J Chem Phys.

[33] Elmounedi N, Bahloul W, Turki M, Amri R, Aoui M, Elbaya W (2022). Impact of needle size on the onset and the progression of disc degeneration in rats. Pain physician.

[34] Agrawal A, Cho SH, Zandi O, Ghosh S, Johns RW, Milliron DJ (2018). Localized surface plasmon resonance in semiconductor nanocrystals. Chem Rev.

[35] He H, Long M, Duan Y, Gu N (2023). Prussian blue nanozymes: progress, challenges, and opportunities. Nanoscale.

[36] Zhang W, Ma D, Du J (2014). Prussian blue nanoparticles as peroxidase mimetics for sensitive colorimetric detection of hydrogen peroxide and glucose. Talanta.

[37] Shen K, Li X, Huang G, Yuan Z, Xie B, Chen T (2023). High rapamycin-loaded hollow mesoporous prussian blue nanozyme targets lesion area of spinal cord injury to recover locomotor function. Biomaterials.

[38] Yu Z, Gong H, Li M, Tang D (2022). Hollow prussian blue nanozyme-richened liposome for artificial neural network-assisted multimodal colorimetric-photothermal immunoassay on smartphone. Biosens Bioelectron.

[39] Wang Y, Cheng H, Wang T, Zhang K, Zhang Y, Kang X (2023). Oxidative stress in intervertebral disc degeneration: Molecular mechanisms, pathogenesis and treatment. Cell Prolif.

[40] Wang Y, Wu Y, Zhang B, Zheng C, Hu C, Guo C (2023). Repair of degenerative nucleus pulposus by polyphenol nanosphere-encapsulated hydrogel gene delivery system. Biomaterials.

[41] Tian Y, Li Y, Liu J, Lin Y, Jiao J, Chen B (2022). Photothermal therapy with regulated Nrf2/NF-κB signaling pathway for treating bacteria-induced periodontitis. Bioact Mater.

[42] Liu Z, Luo Z, Yu H, Zhao E, Guo J, Mou P (2023). Near-infrared light-controlled kartogenin delivery of multifunctional prussian blue nanocomposites for cartilage defect repair. Nanoscale.

[43] Chen Z, Yu Y, Gao Y, Zhu Z (2023). Rational design strategies for nanozymes. ACS nano.

[44] Ye X, Xia L, Yang H, Xu J, Liu T, Wang L (2023). Reactive oxygen/nitrogen species scavenging multi-enzyme mimetic ultrasmall calcium hexacyanoferrate (Ⅲ) nanozyme for hypertension remedy. Mater Today.

[45] Luo C, Yang Q, Lin X, Qi C, Li G (2019). Preparation and drug release property of tanshinone IIA loaded chitosan-montmorillonite microspheres. Int J Biol Macromol.

[46] Wang Y, Che M, Xin J, Zheng Z, Li J, Zhang S (2020). The role of IL-1β and TNF-α in intervertebral disc degeneration. Biomed Pharmacother.

[47] Liu Y, Sun X, Wang L, Dou Y, Tian Y, Yu T (2024). Sequential targeted enzyme-instructed self-assembly supramolecular nanofibers to attenuate intervertebral disc degeneration. Adv Mater.

[48] Molinos M, Almeida CR, Caldeira J, Cunha C, Gonçalves RM, Barbosa MA (2015). Inflammation in intervertebral disc degeneration and regeneration. J R Soc Interface.

[49] Hoyland JA, Le Maitre C, Freemont AJ (2008). Investigation of the role of IL-1 and TNF in matrix degradation in the intervertebral disc. Rheumatology (Oxford).

[50] Le Maitre CL, Hoyland JA, Freemont AJ (2007). Interleukin-1 receptor antagonist delivered directly and by gene therapy inhibits matrix degradation in the intact degenerate human intervertebral disc: an in situ zymographic and gene therapy study. Arthritis Res Ther.

[51] Wu D, Prives C (2018). Relevance of the p53-MDM2 axis to aging. Cell Death Differ.

[52] Huang Y, Che X, Wang PW, Qu X (2024). p53/MDM2 signaling pathway in aging, senescence and tumorigenesis. Semin Cancer Biol.

[53] Meek DW (1994). Post-translational modification of p53. Semin Cancer Biol.

[54] Wang H, Guo M, Wei H, Chen Y (2023). Targeting p53 pathways: mechanisms, structures, and advances in therapy. Signal Transduct Target Ther.

[55] He S, Zhang Z, Luo R, Jiang Q, Yang L, Wang Y (2023). Advances in injectable hydrogel strategies for heart failure treatment. Adv Healthc Mater.

[56] Gutierrez AM, Frazar EM, MV XK, Paul P, Hilt JZ (2022). Hydrogels and hydrogel nanocomposites: Enhancing healthcare through human and environmental treatment. Adv Healthc Mater.

[57] Rennick JJ, Johnston APR, Parton RG (2021). Key principles and methods for studying the endocytosis of biological and nanoparticle therapeutics. Nat Nanotechnol.

[58] Song Y, Wu Y, Xu L, Jiang T, Tang C, Yin C (2021). Caveolae-mediated endocytosis drives robust siRNA delivery of polymeric nanoparticles to macrophages. ACS nano.

[59] Wang SJ, Chen CS, Chen LC (2013). Prussian blue nanoparticles as nanocargoes for delivering DNA drugs to cancer cells. Sci Technol Adv Mater.

[60] Shi Y, Bu W, Chu D, Lin W, Li K, Huang X (2024). Rescuing nucleus pulposus vells from ROS toxic microenvironment via mitochondria-targeted carbon dot-supported prussian blue to alleviate intervertebral disc degeneration. Adv Healthc Mater.

[61] Yang L, Bhujel B, Hou Y, Luo J, An SB, Han I (2023). Effective modulation of inflammation and oxidative stress for enhanced regeneration of intervertebral discs using 3D porous hybrid protein nanoscaffold. Adv Mater.

[62] Zhu Z, Yu Q, Li H, Han F, Guo Q, Sun H (2023). Vanillin-based functionalization strategy to construct multifunctional microspheres for treating inflammation and regenerating intervertebral disc. Bioact Mater.

[63] Chu D, Zhao M, Rong S, Jhe W, Cai X, Xiao Y (2024). Dual-atom nanozyme eye drops attenuate inflammation and break the vicious cycle in dry eye disease. Nanomicro Lett.

[64] Wu H, Xia F, Zhang L, Fang C, Lee J, Gong L (2022). A ROS-sensitive nanozyme-augmented photoacoustic nanoprobe for early diagnosis and therapy of acute liver failure. Adv Mater.

[65] Shi Z, Li X, Chen J, Dai Z, Zhu Y, Wu T (2024). Enzyme-like biomimetic oral-agent enabling modulating gut microbiota and restoring redox homeostasis to treat inflammatory bowel disease. Bioact Mater.

[66] Zhang W, Hu S, Yin JJ, He W, Lu W, Ma M (2016). Prussian blue nanoparticles as multienzyme mimetics and reactive oxygen species scavengers. J Am Chem Soc.

[67] He YJ, Liu XY, Xing L, Wan X, Chang X, Jiang HL (2020). Fenton reaction-independent ferroptosis therapy via glutathione and iron redox couple sequentially triggered lipid peroxide generator. Biomaterials.

[68] Hu C, Xing G, Han W, Hao Y, Zhang C, Zhang Y (2024). Inhibiting demetalation of Fe—N—C via Mn sites for efficient oxygen reduction reaction in zinc-air batteries. Adv Mater.

[69] Liu C, Li M, Li P, Bai Y, Pang J, Fan L (2021). Ruthenium (II)-coordinated supramolecular metallodrug complex realizing oxygen self-supply in situ for overcoming hypoxic tumors. Adv Funct Mater.

[70] Luo T, Yang H, Wang R, Pu Y, Cai Z, Zhao Y (2023). Bifunctional cascading nanozymes based on carbon dots promotes photodynamic therapy by regulating hypoxia and glycolysis. ACS nano.

[71] Liang N, Yuan X, Zhang L, Shen X, Zhong S, Li L (2024). Fatty acid oxidation-induced HIF-1α activation facilitates hepatic urate synthesis through upregulating NT5C2 and XDH. Life Metab.

[72] Wang Y, Kang J, Guo X, Zhu D, Liu M, Yang L (2022). Intervertebral disc degeneration models for pathophysiology and regenerative therapy -benefits and limitations. J Invest Surg.

[73] Shi Y, Li H, Chu D, Lin W, Wang X, Wu Y (2023). Rescuing nucleus pulposus cells from senescence via dual-functional greigite nanozyme to alleviate intervertebral disc degeneration. Adv Sci (Weinh).

[74] Chen L, Shen Q, Liu Y, Zhang Y, Sun L, Ma X (2025). Homeostasis and metabolism of iron and other metal ions in neurodegenerative diseases. Signal Transduct Target Ther.

[75] Wang M, Yan C, Li X, Yang T, Wu S, Liu Q (2024). Non-invasive modulation of meningeal lymphatics ameliorates ageing and Alzheimer's disease-associated pathology and cognition in mice. Nat Commun.

