# Supplementary material for: Tailorable bimetallic nanozyme mitigates intervertebral disc degeneration by inhibiting oxidative stress and inflammageing
Source: Theranostics. 2025 Jun 9;15(14):6957–82. doi: 10.7150/thno.108592 (PMC12203817; doi:10.7150/thno.108592)
Supplement: Supplementary file 1 — Supplementary figures and tables. [file thnov15p6957s1.pdf]

Supporting information

Tailorable bimetallic nanozyme mitigates intervertebral disc degeneration by inhibiting oxidative stress and inflammageing

Anran Zhang[1], Haiyang Gao[1], Xianglong Chen[1], Pengzhi Shi, Zhangrong Cheng, Yuhang Chen, Wang Wu, Wenbo Wu, Cao Yang, Yukun Zhang[*]

Affiliation: Department of Orthopedics, Union Hospital, Tongji Medical College, Huazhong University of Science and Technology, Wuhan 430022, China

1: These authors contributed equally to this work.

*: Corresponding author: Yukun Zhang

E-mail: zykunion@hust.edu.cn

Supplemental figures:

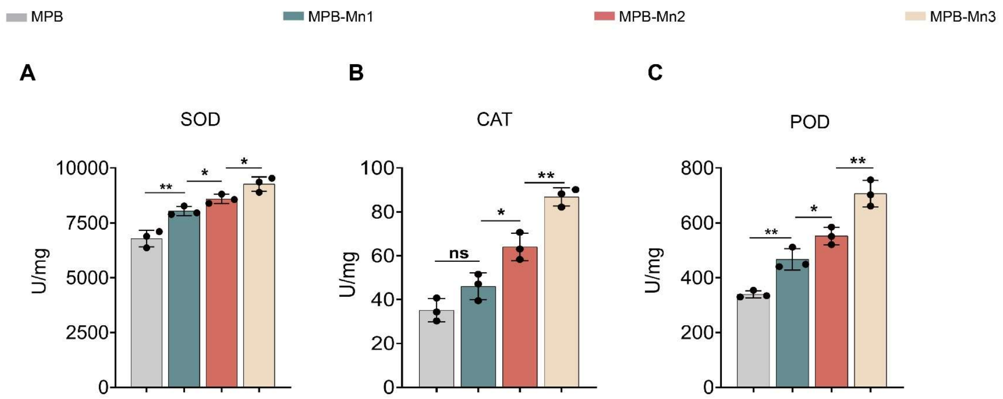

Figure S1: Specific activity measurements of multiple enzyme-like activities in each group. (A) SOD. (B) CAT. (C) POD. MPB group: NPCs treated with MPB; MPB-Mn1 group: NPCs treated with MPB-Mn1; MPB-Mn2 group: NPCs treated with MPB-Mn2; MPB-Mn3 group: NPCs treated with MPB-Mn3 (n =3, mean ± SD). (* means P < 0.05 and ** means P < 0.01, ns means no significant difference).

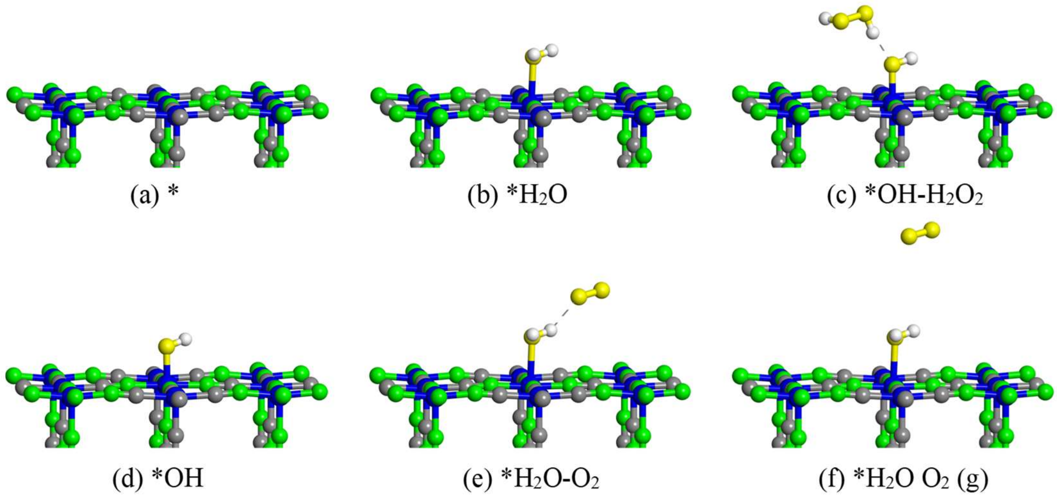

Figure S2: The optimized structures of the intermediates of the SOD path on FeN_4 site of MPB (200) surface.

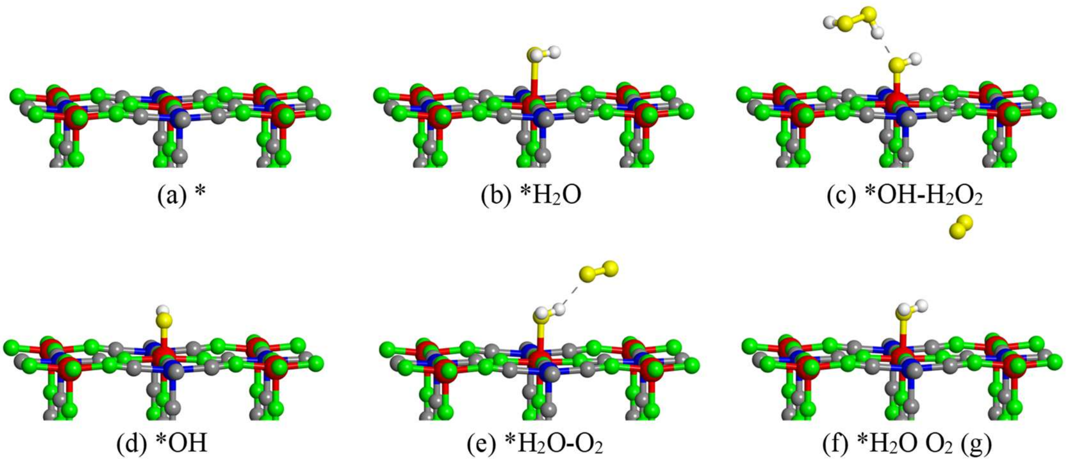

Figure S3: The optimized structures of the intermediates of the SOD path on MnN_4 site of MPB-Mn (200) surface.

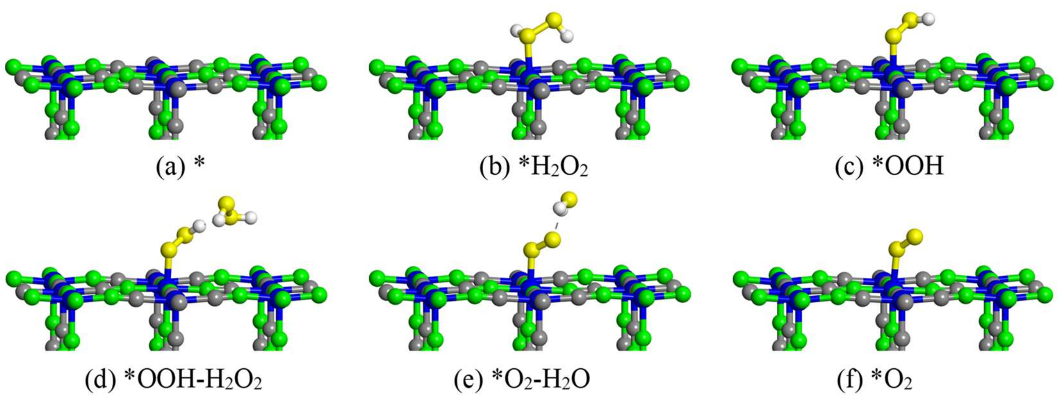

Figure S4: The optimized structures of the intermediates of the CAT path on FeN_4 site of MPB (200) surface.

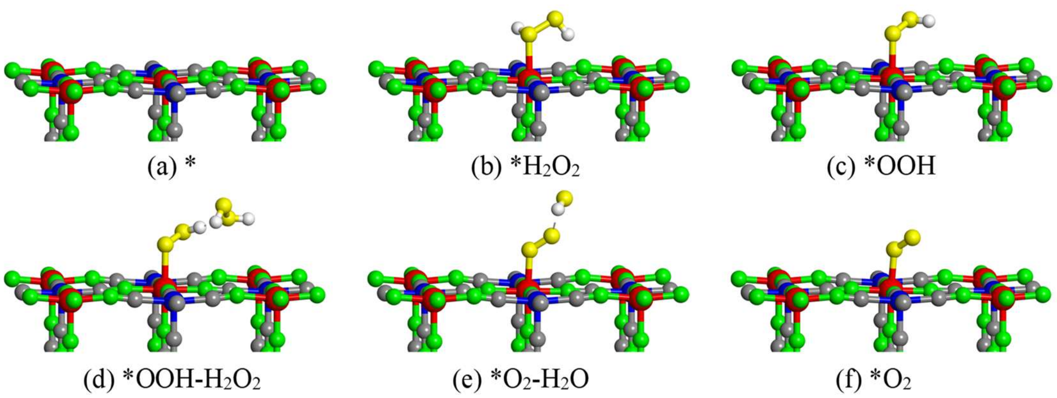

(a) * (b) $*H_2O_2$ (c) *OOH

(d) $*OOH-H_2O_2$ (e) $*O_2-H_2O$ (f) $*O_2$

Figure S5: The optimized structures of the intermediates of the CAT path on MnN_4 site of MPB-Mn (200) surface.

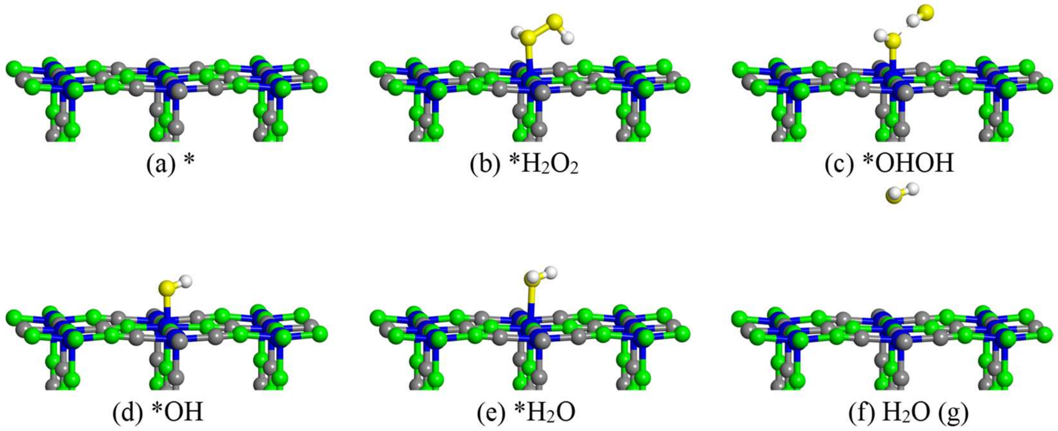

Figure S6: The optimized structures of the intermediates of the POD path on FeN_4 site of MPB (200) surface.

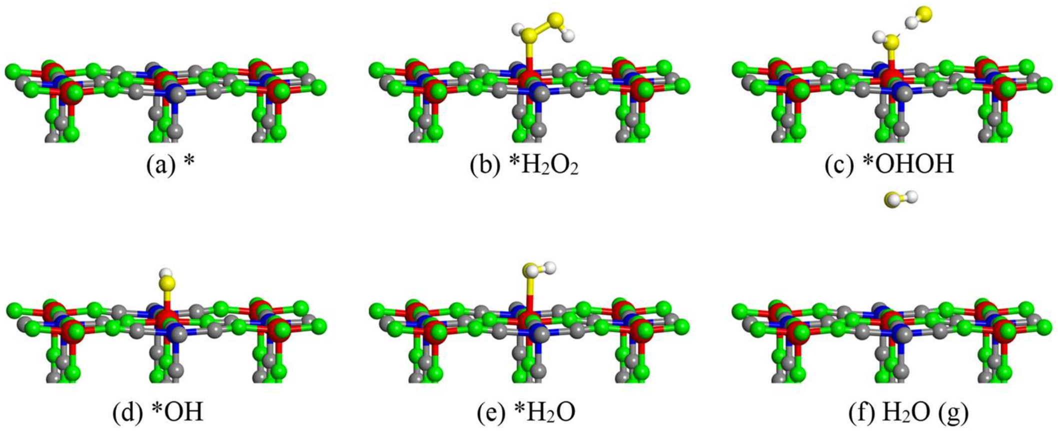

Figure S7: The optimized structures of the intermediates of the POD path on MnN_4 site of MPB-Mn (200) surface.

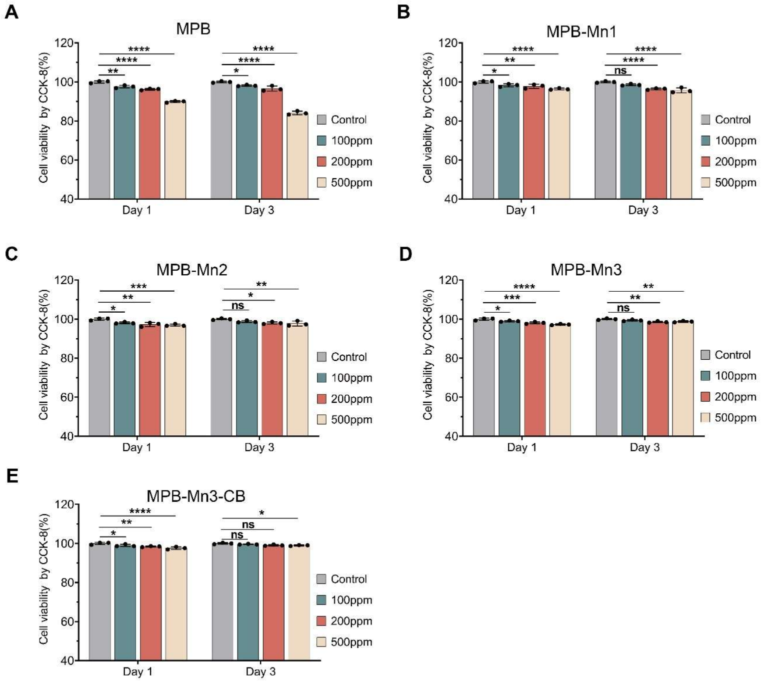

Figure S8: Establishment of ultimate utilization concentrations for each material group (A) CCK-8 analysis of different concentrations of MPB at 1, 3 days (n =3, mean ± SD). (B) CCK-8 analysis of different concentrations of MPB-Mn1 at 1, 3 days (n =3, mean ± SD). (C) CCK-8 analysis of different concentrations of MPB-Mn2 at 1, 3 days (n =3, mean ± SD). (D) CCK-8 analysis of different concentrations of MPB-Mn3 at 1, 3 days (n =3, mean ± SD). (E) CCK-8 analysis of different concentrations of MPB-Mn3-CB at 1, 3 days (n =3, mean ± SD). (* means $P < 0.05$, ** means $P < 0.01$, *** means $P < 0.001$ and **** means $P < 0.0001$, ns means no significant difference).

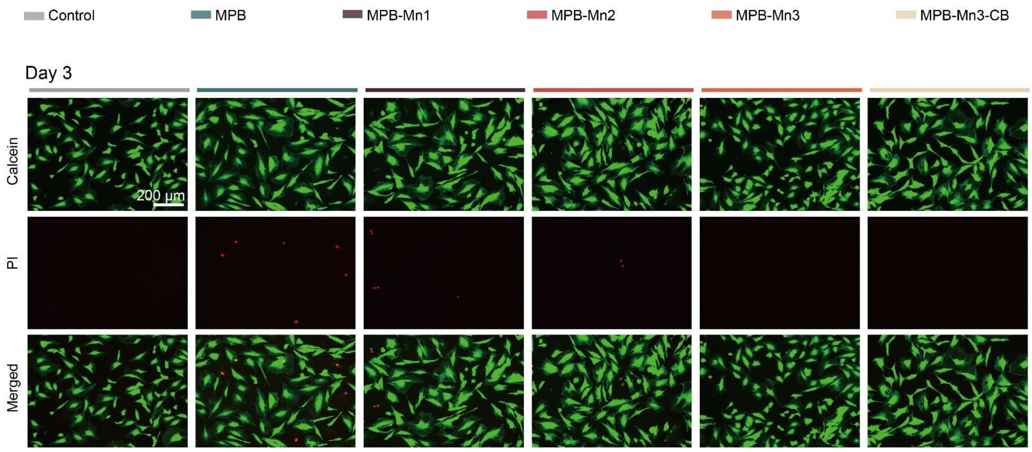

Figure S9: Representative Live/Death staining images of the six groups at Day 3. Control group: normal NPCs; MPB group: NPCs treated with MPB; MPB-Mn1 group: NPCs treated with MPB-Mn1; MPB-Mn2 group: NPCs treated with MPB-Mn2; MPB-Mn3 group: NPCs treated with MPB-Mn3; MPB-Mn3-CB group: NPCs treated with MPB-Mn3-CB. Scale bar: 200 μm.

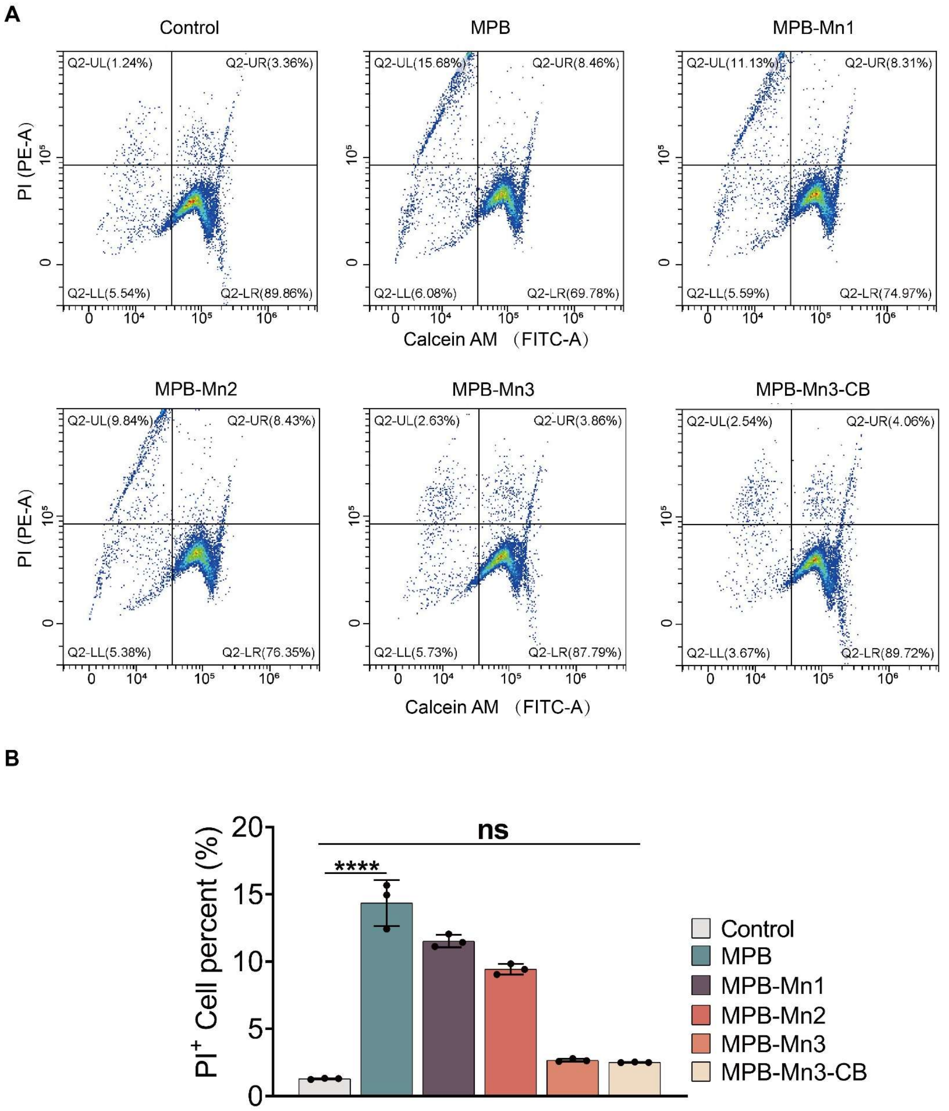

Figure S10: Live/Death staining and proportion of PI^+ cell percent in NPCs at Day7 under various treatment conditions assessed by flow cytometry. Control group: normal NPCs; MPB group: NPCs treated with MPB; MPB-Mn1 group: NPCs treated with MPB-Mn1; MPB-Mn2 group: NPCs treated with MPB-Mn2; MPB-Mn3 group: NPCs treated with MPB-Mn3; MPB-Mn3-CB group: NPCs treated with MPB-Mn3-CB. (A) Live/Death staining in NPCs at Day7 under various treatment conditions. (B) PI^+ cell percent in NPCs at Day7 under various treatment conditions (n =3, means ± SD). (**** means $P < 0.0001$, ns means no significant difference).

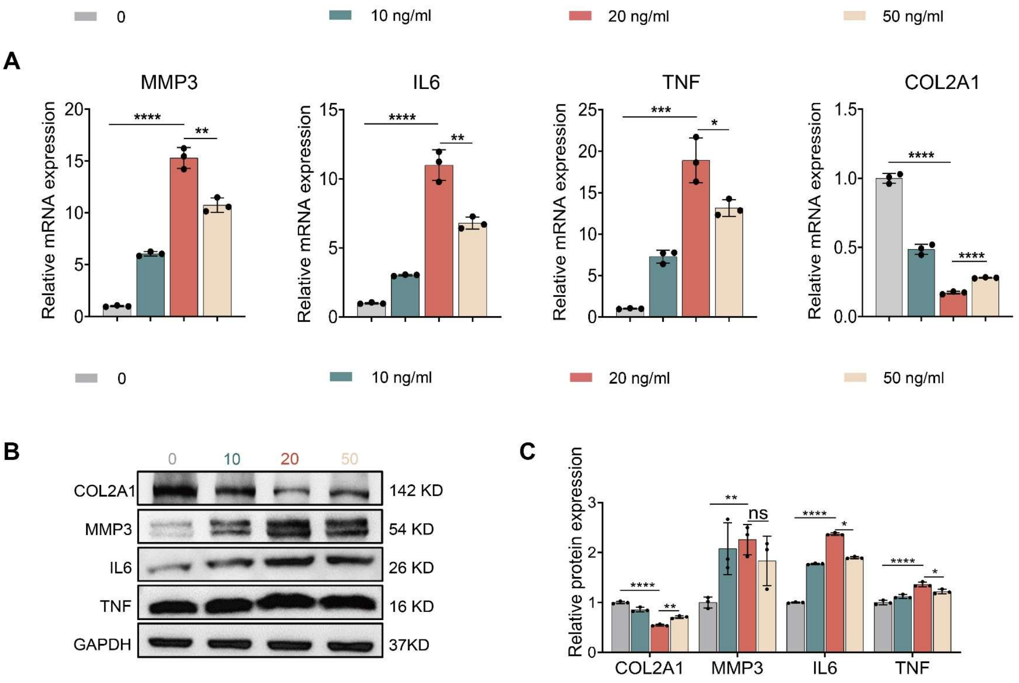

Figure S11: Establishment of IDD cell model. (A) RT-qPCR analysis of MMP3, IL6, TNF, and COL2A1 mRNA expression at IL1B concentrations of 0, 10 ng/ml, 20 ng/ml, and 50 ng/ml (n =3, mean ± SD). (B, C) Protein expression levels and quantitative analysis of COL2A1, MMP3, IL6, and TNF in NPCs under different treatment conditions (n =3, mean ± SD). (* means P < 0.05, ** means P < 0.01 and **** means P < 0.0001, ns means no significant difference).

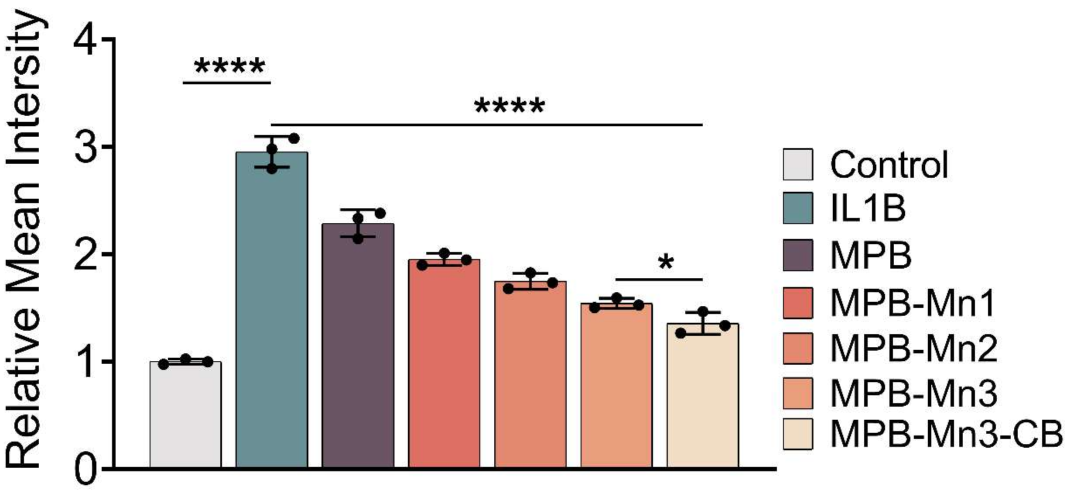

Figure S12: Relative fluorescence intensity of ROS levels in NPCs of the seven groups by flow cytometry. Control group: normal NPCs; IL1B group: NPCs treated with IL1B; MPB group: NPCs treated with IL1B and MPB; MPB-Mn1 group: NPCs treated with IL1B and MPB-Mn1; MPB-Mn2 group: NPCs treated with IL1B and MPB-Mn2; MPB-Mn3 group: NPCs treated with IL1B and MPB-Mn3; MPB-Mn3-CB group: NPCs treated with IL1B and MPB-Mn3-CB (n =3, mean ± SD). (* means $P < 0.05$ and **** means $P < 0.0001$).

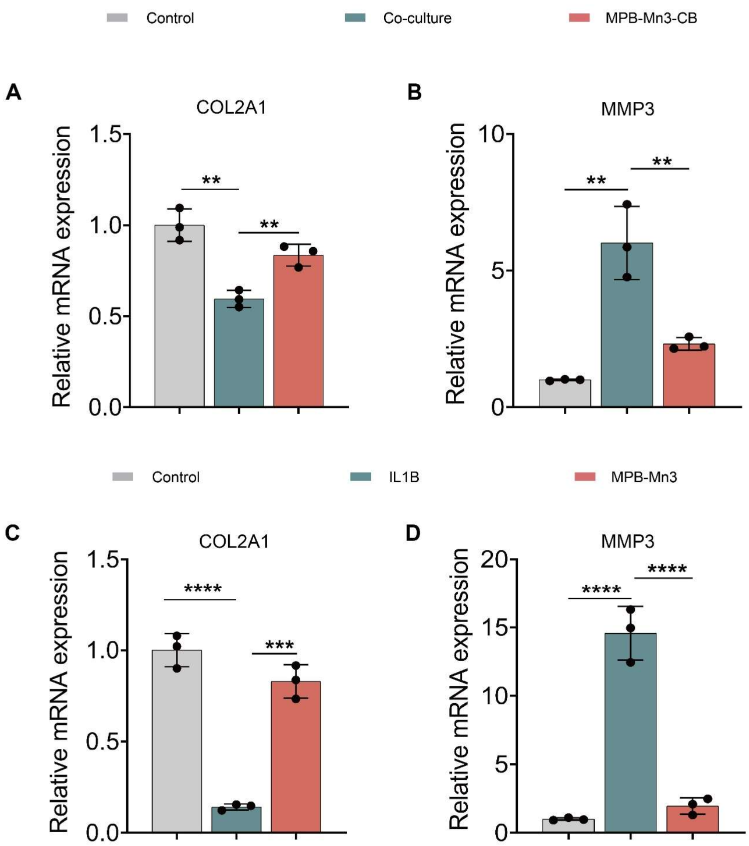

Figure S13: Quantitative analysis of relative mRNA expression levels of co-culture inflammation model group and the IL1B inflammation model group. (A， B) RT-qPCR analysis of COL2A1 and MMP3 mRNA expression. Control group: normal NPCs; Co-culture group: NPCs treated with LPS-activated immune cells; MPB-Mn3-CB group: NPCs treated with LPS-activated immune cells and MPB-Mn3-CB. (C, D) RT-qPCR analysis of COL2A1 and MMP3 mRNA expression. Control group: normal NPCs; IL1B group: NPCs treated with IL1B; MPB-Mn3-CB group: NPCs treated with IL1B and MPB-Mn3-CB (n =3, mean ± SD). (** means $P < 0.01$, *** means $P < 0.001$ and **** means $P < 0.0001$).

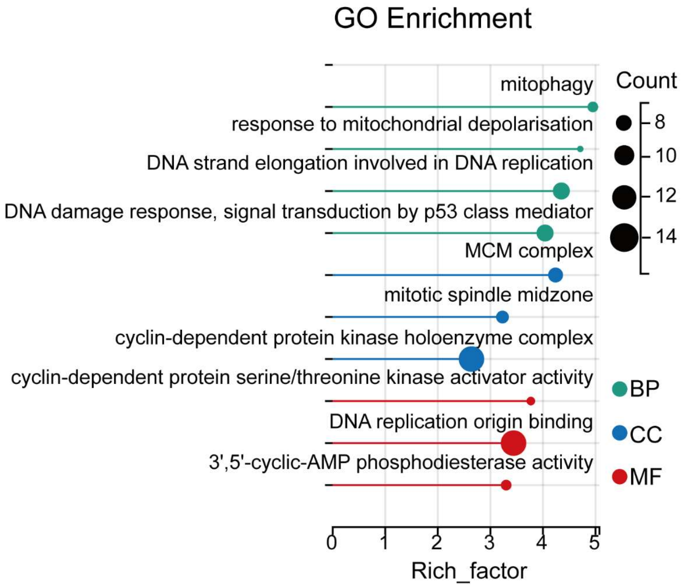

Figure S14: GO pathway enrichment analysis of DEGs.

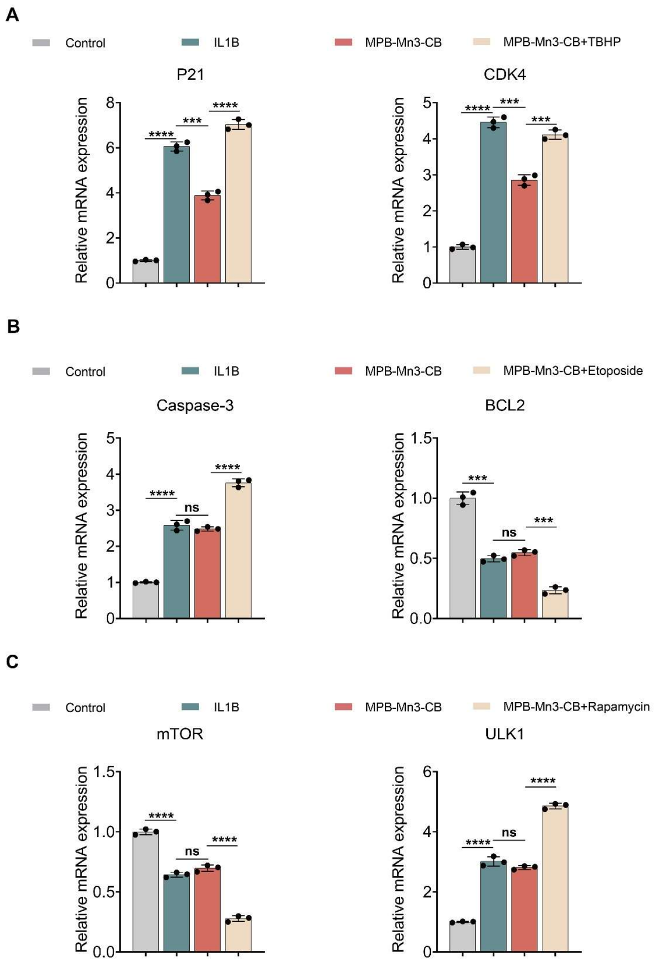

Figure S15: Quantitative analysis of relative mRNA expression levels in NPCs across the four groups by RT-qPCR. (A) RT-qPCR analysis of P21, CDK4 mRNA expression. Control group: normal NPCs; IL1B group: NPCs treated with IL1B; MPB-Mn3-CB group: NPCs treated with IL1B and MPB-Mn3-CB; MPB-Mn3-CB+TBHP group: NPCs treated with IL1B and MPB-Mn3-CB and TBHP. (B) RT-qPCR analysis of Caspase-3, BCL2 mRNA expression. Control group: normal NPCs; IL1B group: NPCs treated with IL1B; MPB-Mn3-CB group: NPCs treated with IL1B and MPB-Mn3-CB; MPB-Mn3-CB+ Etoposide group: NPCs treated with IL1B and MPB-Mn3-CB and Etoposide. (C) RT-qPCR analysis of mTOR, ULK1 mRNA expression. RT-qPCR analysis of Caspase-3, BCL2 mRNA expression. Control group: normal NPCs; IL1B group: NPCs treated with IL1B; MPB-Mn3-CB group: NPCs treated with IL1B and MPB-Mn3-CB; MPB-Mn3-CB+ Rapamycin group: NPCs treated with IL1B and MPB-Mn3-CB and Rapamycin (n =3, mean ± SD). (*** means $P < 0.001$ and **** means $P < 0.0001$, ns means no significant difference).

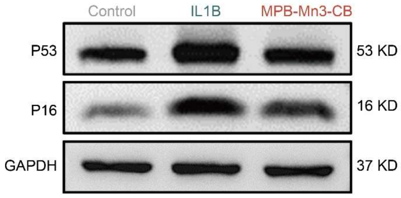

Figure S16: Protein expression levels of P16 and P53 in NPCs under various treatment conditions Control group: normal NPCs; IL1B group: NPCs treated with IL1B; MPB-Mn3-CB group: NPCs treated with IL1B and MPB-Mn3-CB (n = 3, means ± SD).

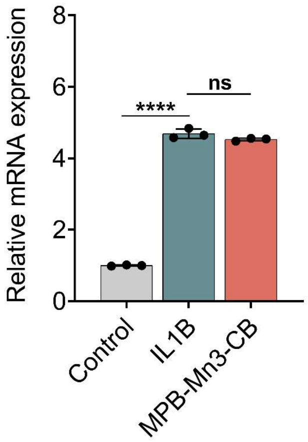

Figure S17: RT-qPCR analysis for P53 mRNA expression of the three groups. Control group: normal NPCs; IL1B group: NPCs treated with IL1B; MPB-Mn3-CB group: NPCs treated with IL1B and MPB-Mn3-CB (n = 3, means ± SD). (**** means $P < 0.0001$, ns means no significant difference).

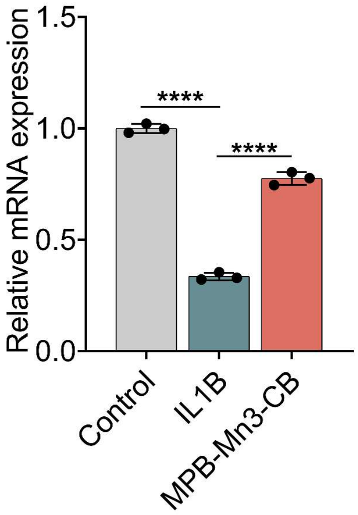

Figure S18: RT-qPCR analysis for MDM2 mRNA expression of the three groups. Control group: normal NPCs; IL1B group: NPCs treated with IL1B; MPB-Mn3-CB group: NPCs treated with IL1B and MPB-Mn3-CB (n = 3, means ± SD). (**** means $P < 0.0001$).

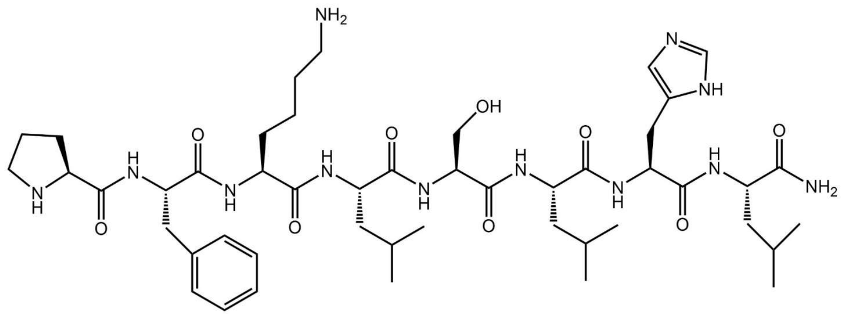

Figure S19: Structural formula of Jelleine-1.

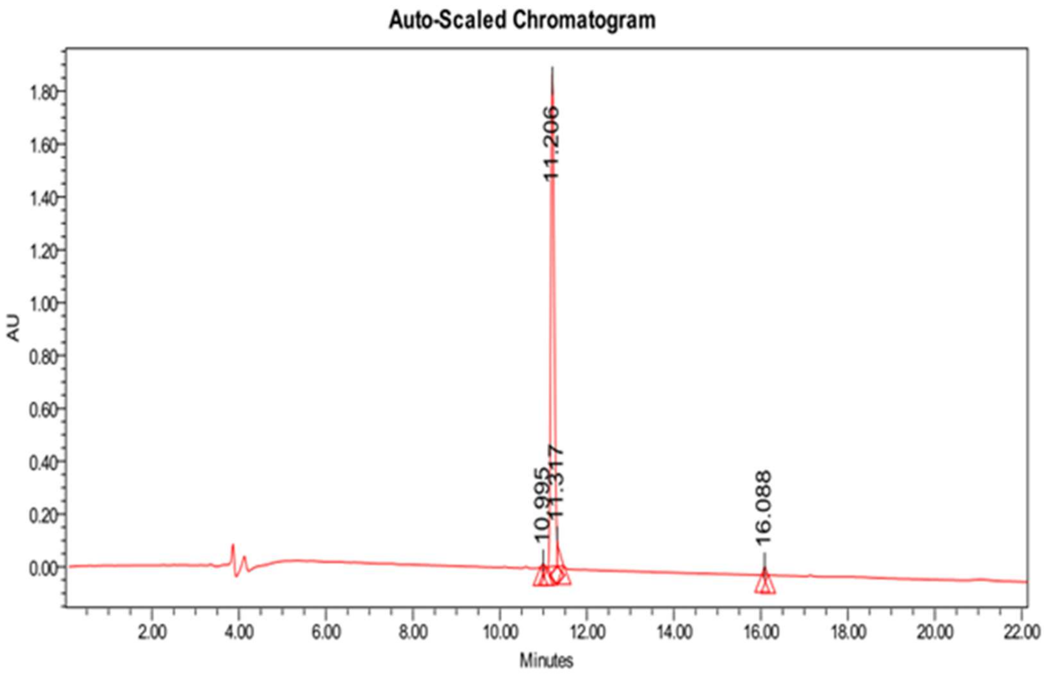

Peak Results

	RT	Area	Height	% Area
1	10.995	47680	11640	0.44
2	11.206	10382018	1871005	95.08
3	11.317	375984	96549	3.44
4	16.088	11301[illegible]	27294	1.04

Figure S20: HPLC Analysis Report.

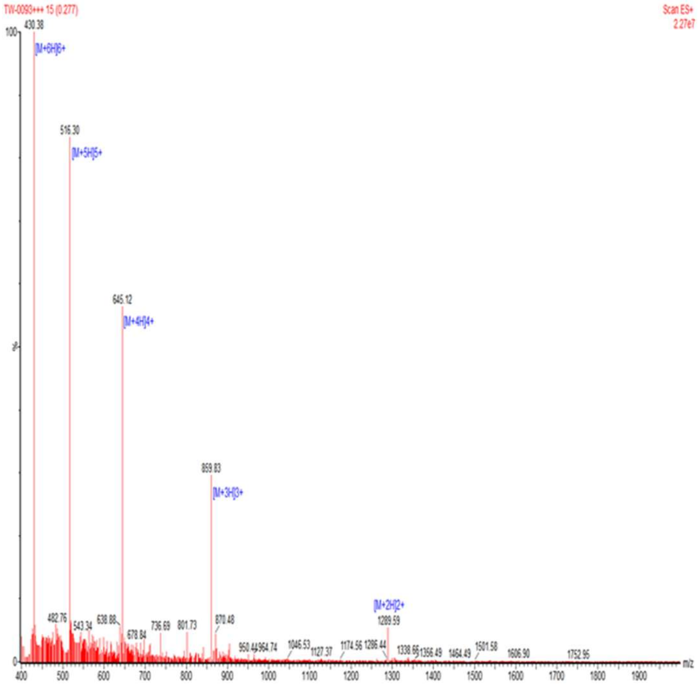

Figure S21: MS Analysis Report.

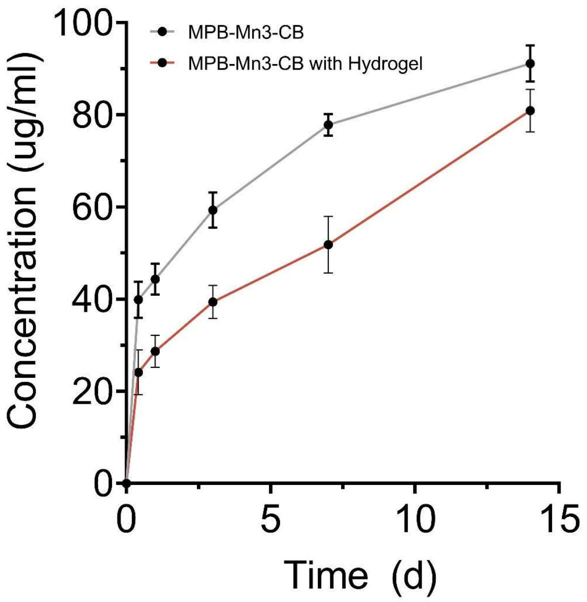

Figure S22: Comparison of release profiles of MPB-Mn3-CB with and without hydrogel encapsulation (n =3, mean ± SD).

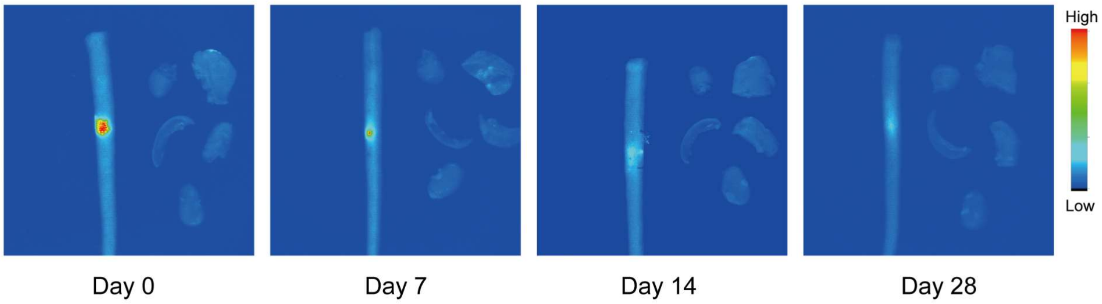

Figure S23: In vivo fluorescence imaging of IVD and major organs of SD rats at different time intervals after injection of Cy5.5-labeled nanozymes.

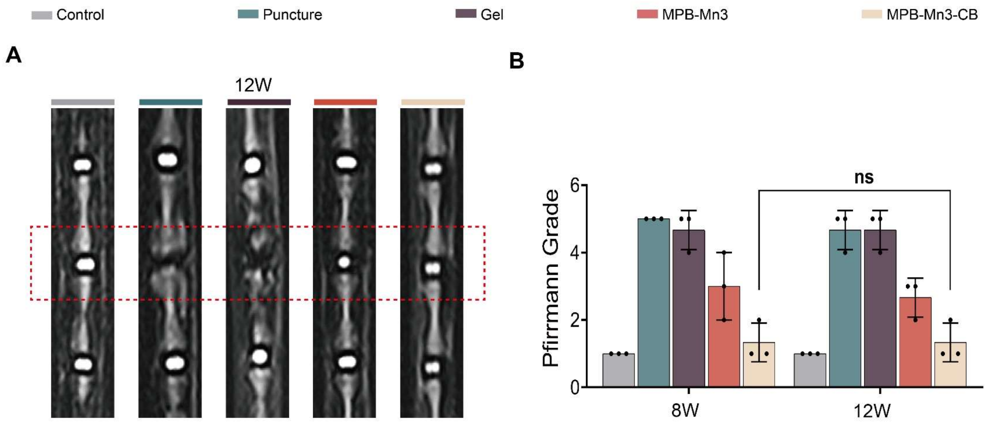

Figure S24: (A) Representative 12W MRI images of each group. (B) Quantitative of Pfirrmann grades of 8 and 12 weeks. Control group: normal intervertebral disc; Puncture group: punctured intervertebral disc; Gel group: punctured intervertebral disc treated with Gel; MPB-Mn3 group: punctured intervertebral disc treated with Gel-MPB-Mn3 hydrogel; MPB-Mn3-CB group: punctured intervertebral disc treated with Gel-MPB-Mn3-CB hydrogel (n =3, mean ± SD). (ns means no significant difference).

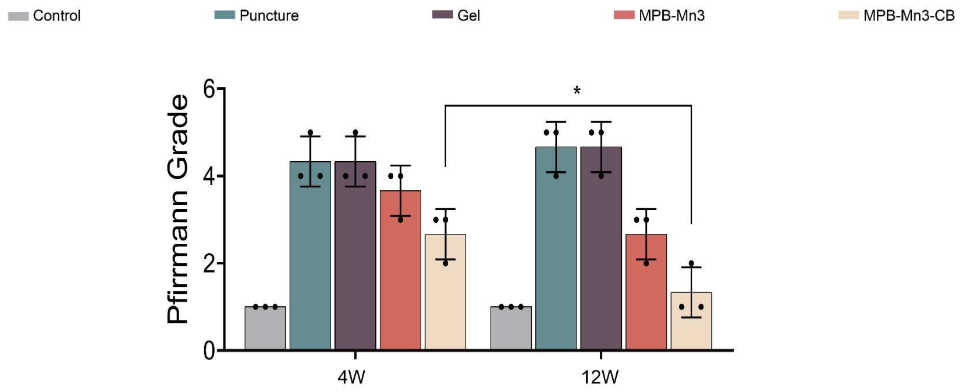

Figure S25: Quantitative of Pfirrmann grades of 4 and 12 weeks. Control group: normal intervertebral disc; Puncture group: punctured intervertebral disc; Gel group: punctured intervertebral disc treated with Gel; MPB-Mn3 group: punctured intervertebral disc treated with Gel-MPB-Mn3 hydrogel; MPB-Mn3-CB group: punctured intervertebral disc treated with Gel-MPB-Mn3-CB hydrogel (n =3, mean ± SD). (* means $P < 0.05$).

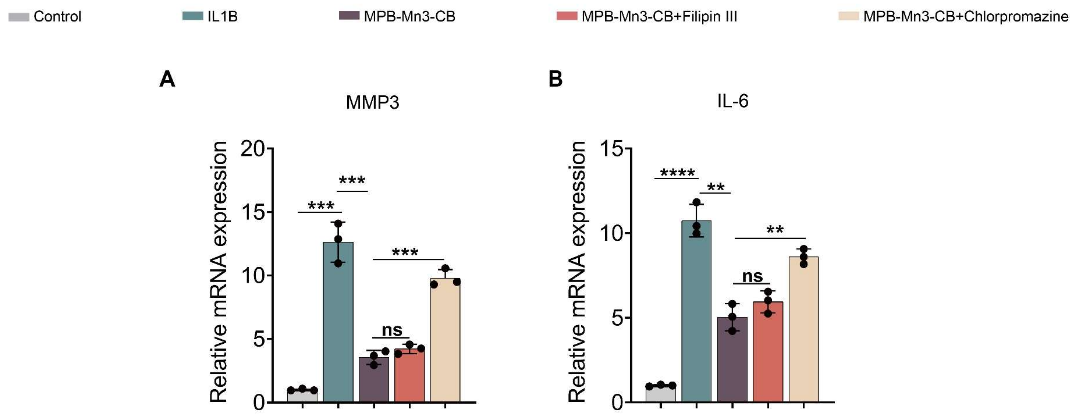

Figure S26: Quantitative analysis of relative mRNA expression levels in NPCs across the five groups by RT-qPCR. Control group: normal NPCs; IL1B group: NPCs treated with IL1B; MPB-Mn3-CB group: NPCs treated with IL1B and MPB-Mn3-CB; MPB-Mn3-CB+ Filipin III group: NPCs treated with IL1B and MPB-Mn3-CB and Filipin III； MPB-Mn3-CB+ Chlorpromazine group: NPCs treated with IL1B and MPB-Mn3-CB and Chlorpromazine. (A) RT-qPCR analysis of MMP3 mRNA expression. (B) RT-qPCR analysis of IL-6 mRNA expression (n =3, mean ± SD). (** means $P < 0.01$, *** means $P < 0.001$ and **** means $P < 0.0001$).

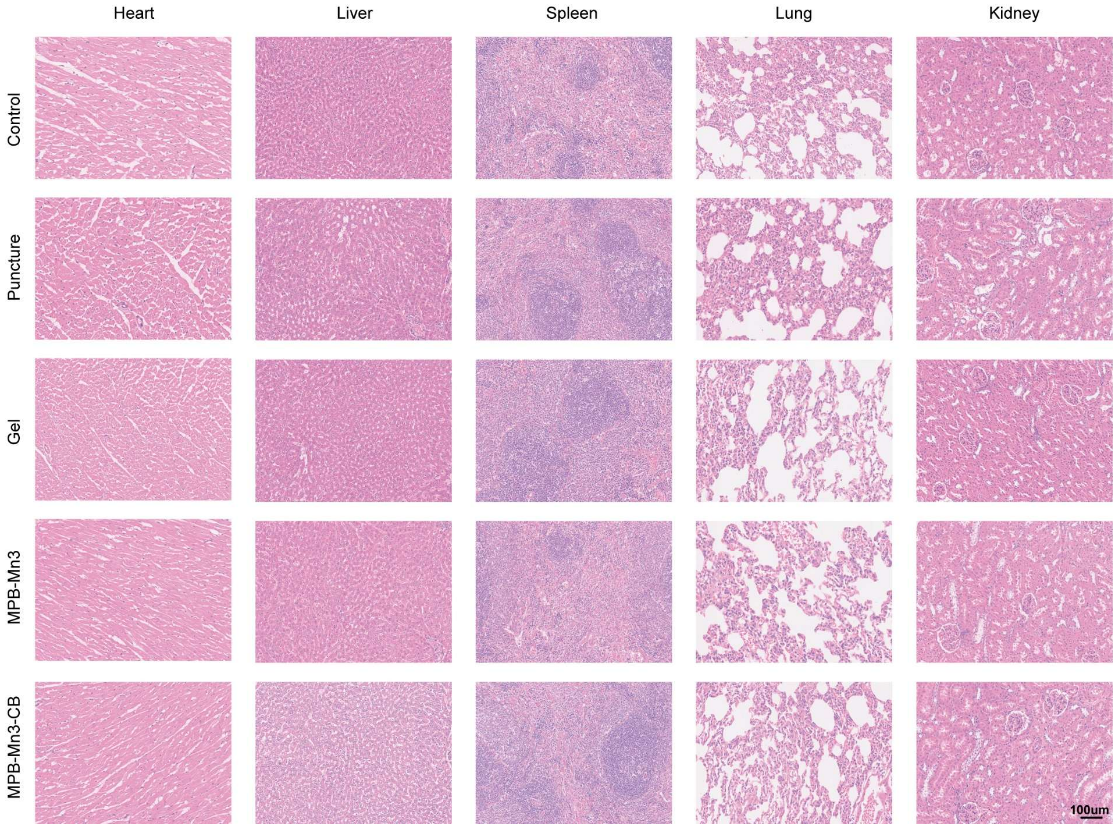

Figure S27: Histological sections of the heart, liver, spleen, lungs, and kidneys in vivo in Control, Puncture, Gel, MPB-Mn3, and MPB-Mn3-CB groups at 8 weeks. Control group: normal intervertebral disc; Puncture group: punctured intervertebral disc; Gel group: punctured intervertebral disc treated with Gel; MPB-Mn3 group: punctured intervertebral disc treated with Gel-MPB-Mn3 hydrogel; MPB-Mn3-CB group: punctured intervertebral disc treated with Gel-MPB-Mn3-CB hydrogel. Scale bar: 100 μm.

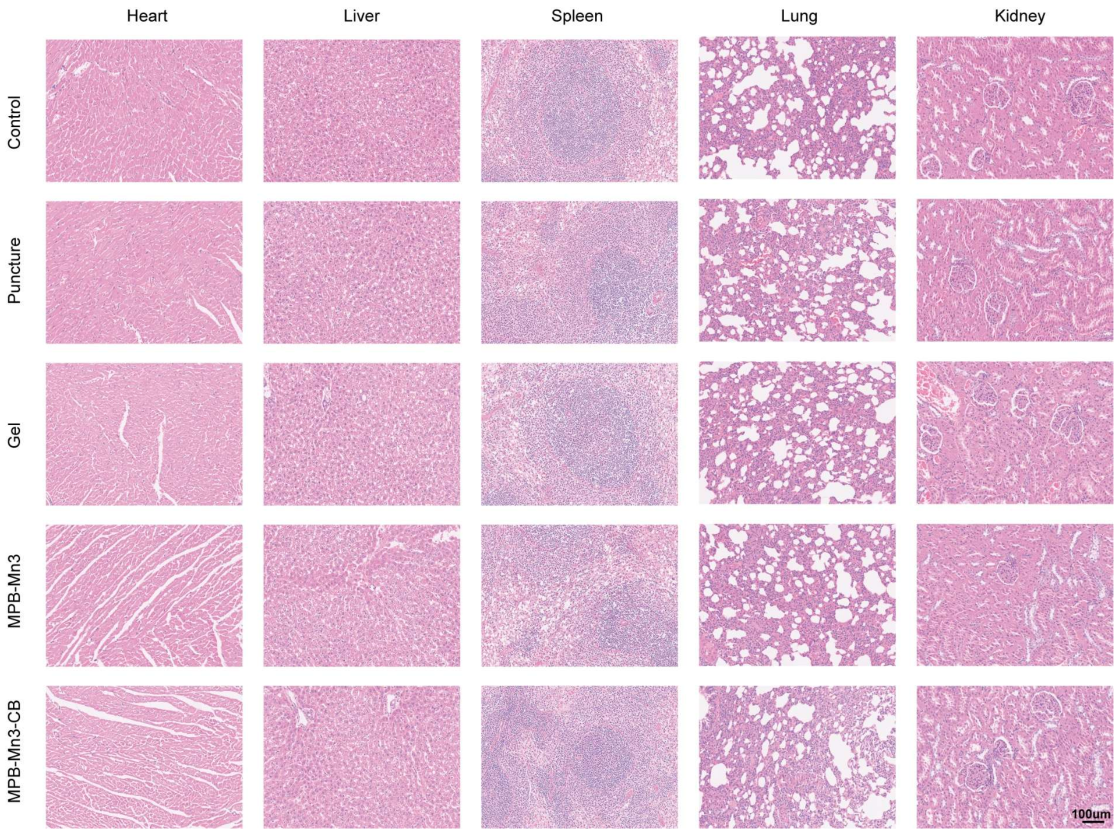

Figure S28: Histological sections of the heart, liver, spleen, lungs, and kidneys in vivo in Control, Puncture, Gel, MPB-Mn3, and MPB-Mn3-CB groups at 12 weeks. Control group: normal intervertebral disc; Puncture group: punctured intervertebral disc; Gel group: punctured intervertebral disc treated with Gel; MPB-Mn3 group: punctured intervertebral disc treated with Gel-MPB-Mn3 hydrogel; MPB-Mn3-CB group: punctured intervertebral disc treated with Gel-MPB-Mn3-CB hydrogel. Scale bar: 100 μm.

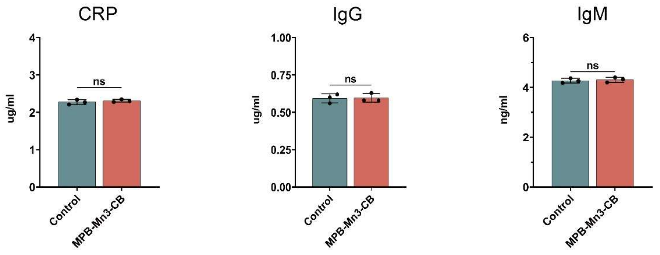

Figure S29: Immunogenicity testing of MPB-Mn3-CB on the seventh day after injection. (A-C) CRP, C-reactive protein. IgG, Immunoglobulin G. IgM, Immunoglobulin M (n =3, mean ± SD). (ns means no significant difference).

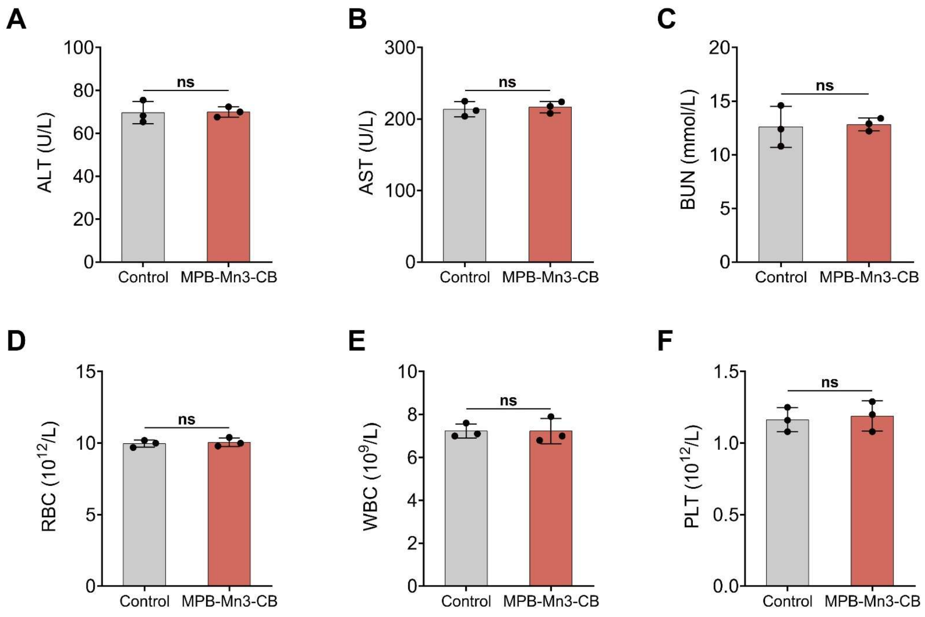

Figure S30: Blood biochemistry and routine blood tests. (A-C) ALT, alanine aminotransferase. AST, aspartic acid transferase. BUN, blood urea nitrogen. (D-F) Hematology examination analysis. RBC, red blood cells. WBC, white blood cells. PLT, blood platelet (n =3, mean ± SD). (ns means no significant difference).

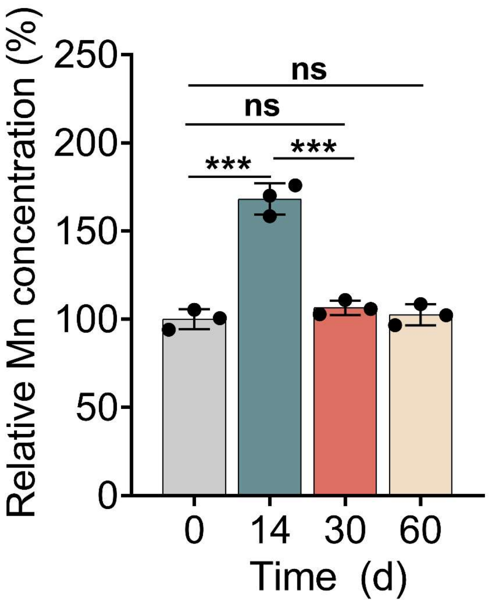

Figure S31: Relative concentration of Mn in the IVD of MPB-Mn3-CB rat were measured by ICP-MS at 0,14, 30 60 d after injection (n =3, mean ± SD). (*** means P < 0.001 and ns means no significant difference).

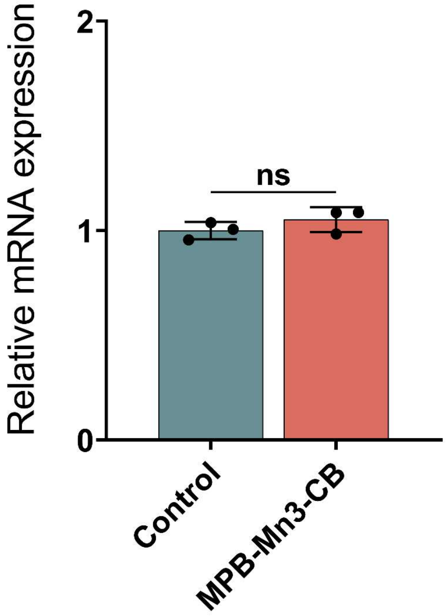

Figure S32: RT-qPCR analysis for Hif-1α mRNA expression of the two groups. Control group: normal NPCs; MPB-Mn3-CB group: NPCs treated with MPB-Mn3-CB (n = 3, means ± SD). (ns means no significant difference

Supplemental tables:

Table S1: Energy change of the SOD reaction path on FeN_4 site of MPB (200) and MnN_4 site of MPB-Mn (200) surfaces

	E (FeN_4, eV)	ΔE	PlotE	E (MnN_4, eV)	ΔE	PlotE
surface	-640.14376424	0.00000000	0.00000000	-650.8932075	0.00000000	0.00000000
$*H_2O$	-654.43221495	-0.06493670	-0.06493670	-665.4399309	-0.32320933	-0.32320933
$*OH\text{-}H_2O_2$	-668.18363045	-0.08369070	-0.14862740	-679.6558027	-0.54814702	-0.87135635
*OH	-649.73632831	0.32957734	0.18094994	-661.2577791	0.28029875	-0.59105760
$*H_2O\text{-}O_2$	-664.32313237	-0.91907926	-0.73812932	-675.5993361	-0.67383217	-1.26488977
$H_2O(g)$	-14.22351401	-0.03290379	-0.77103311	-14.2235140	-0.30139161	-1.56628138
OOH^-	-13.66772480			-13.6677248		
$H_2O_2(g)$	-18.11772480			-18.1177248		
$O_2(g)$	-9.85801363			-9.8580136		

Table S2: Energy change of the CAT reaction path on FeN_4 site of MPB (200) and MnN_4 site of MPB-Mn (200) surfaces

	E (FeN_4, eV)	ΔE	PlotE	E (MnN_4, eV)	ΔE	PlotE
surface	-640.14376424	0.00000000	0.00000000	-650.89320752	0.00000000	0.00000000
*H_2O_2	-658.46296600	-0.20147696	-0.20147696	-669.55728455	-0.54635223	-0.54635223
*OOH	-654.05241016	1.00852167	0.80704471	-665.21438500	0.94086538	0.39451315
*OOH-H_2O_2	-672.55048702	-0.38035206	0.42669265	-683.65366320	-0.32155340	0.07295975
*O_2-H_2O	-664.17143472	0.79432088	1.22101353	-675.28371118	0.78522060	0.85818035
*O_2	-650.48712485	-0.53920414	0.68180939	-660.85980862	0.20038855	1.05856890
H_2O(g)	-14.22351401	0.48534698	1.16715637	-14.22351401	0.10858747	1.16715637
H_2(g)	-6.80406834			-6.80406834		
OH^-	-7.58473142			-7.58473142		
H_2O_2(g)	-18.11772480			-18.11772480		
O_2(g)	-9.85801363			-9.85801363		

Table S3: Energy change of the POD reaction path on FeN_4 site of MPB (200) and MnN_4 site of MPB-Mn (200) surfaces

	E (FeN_4, eV)	ΔE	PlotE	E (MnN_4, eV)	ΔE	PlotE
surface	-640.14376424	0	0	-650.8932075	0	0
*H_2O_2	-658.46296600	-0.20147696	-0.20147696	-669.5572846	-0.54635223	-0.54635223
*OHOH	-657.74910523	0.71386077	0.51238381	-669.2784568	0.27882772	-0.26752451
*OH	-649.73632831	-2.93869493	-2.29631911	-661.2577791	-2.80080214	-3.06832665
*H_2O	-654.36992473	-1.23156225	-3.52788136	-665.4399309	-0.78011756	-3.84844421
H_2O(g)	-14.22351401	0.00264648	-3.52523488	-14.22351401	0.32320933	-3.52523488
H_2(g)	-6.80406834			-6.80406834		
H_2O_2(g)	-18.11772480			-18.1177248		

Table S4: Information of antibodies

Antibodies	Source	Catalog number
COL2A1	Affinity	AF0135
P53	Proteintech	60283-2-Ig
P16	Proteintech	10883-1-AP
TNF	Proteintech	60291-1-Ig
GAPDH	Proteintech	10494-1-AP
IL6	Zenbio	381207
MMP3	Zenbio	380816
MDM2	Zenbio	310329
Ubiquitin	Proteintech	10201-2-AP

Table S5: Primers of targeted genes

Gene name	Forward (5’→3’)	Reverse (5’→3’)
IL6	CAGCCACTCACCTCTTCAGA	ACCAGGCAAGTCTCCTCATT
TNF	ACTGAACTTCGGGGTGATCG	GCTTGGTGGTTTGCTACGAC
MMP3	AATCCTACTGTTGCTGTGCG	CATCACCTCCAGAGTGTCGG
COL2A1	TGGACGATCAGGCGAAACC	GCTGCGGATGCTCTCAATCT
GAPDH	GGGCTGCTTTTAACTCTGGT	TGATTTTGGAGGGATCTCGC
MDM2	CAGTAGCAGTGAATCTACAGGGA	CTGATCCAACCAATCACCTGAAT
p53	CAGCACATGACGGAGGTTGT	TCATCCAAATACTCCACACGC
p21	TGTCCGTCAGAACCCATGC	AAAGTCGAAGTTCCATCGCTC
BCL-2	GACTTTGCAGAGATGTCCAG	TCAGGTACTCAGTCATCCAC
mTOR	CCATGGAACTCCGAGAGATGAG	GCAAATCTGCCAATTCGGGT
Caspase-3	*ATGGAAGCGAATCAATGGACTCT*	CTGCATCGACATCTGTACCAGA
ULK1	*CCACCCAGTTCCAAACACCT*	CCAACTTGAGGAGATGGCGT

HIF1A	*CCAGCAGACTCAAATACAAGAACC*	TGTATGTGGGTAGGAGATGGAGAT

Table S6: Pfirrmann classification system

Grade	Structure	Distinction of Nucleus and Anulus	Signal Intensity	Height of Intervertebral Disc
Ⅰ	Homogeneous, bright white	Clear	Hyperintense, isointense to cerebrospinal fluid	Normal
Ⅱ	Inhomogeneous with or without horizontal bands	Clear	Hyperintense, isointense to cerebrospinal fluid	Normal
Ⅲ	Inhomogeneous, gray	Unclear	Intermediate;	Normal to slightly decreased
Ⅳ	Inhomogeneous, gray to black	Lost	Intermediate to hypointense	Normal to moderately decreased
Ⅴ	Inhomogeneous, black	Lost	Hyperintense	Collapsed disc space

Table S7: Histological grading scores (H&E) of AF and NP

Scores[a]	AF	NP
0	Well-organized fibrous lamellae without ruptured or serpentine fibers	Normal cellularity with large vacuoles and stellar- shaped nucleus consistently dispersed in the nucleus
1	Ruptured or serpentine fibers in less than 30% of the annulus	Slight decrease in the number of cells (<50%) with/ Without cell clustering
2	Ruptured or serpentine fibers in more than 30% of the annulus with reversed contour	Moderate to severe decrease in the number of cells (> 50%) with mostly no vacuolization and occupied by proliferative CNT (<50% of nucleus area)
3	Indistinct and disorganized annulus	Severe replacement by proliferative CNT (>50% of nucleus area) with small area of vacuole cells

H&E: hematoxylin and eosin; AF: annulus fibrosus; NP: nucleus pulposus; CNT: connective tissue.

Scores[a] range from a normal disc (0 point) to severely degenerated disc (6 points).

Table S8: Histological grading system of intervertebral disc

Category	Score
Morphology of the NP	Score 0: round shape and the NP constitutes >75% of the disc area;<br>Score 1: round shape and the NP constitutes 50–75% of the disc area;<br>Score 2: round shape and the NP constitutes 25–50% of the disc area;<br>Score 3: round shape and the NP constitutes <25% of the disc area.
Cellularity of the NP	Score 0: stellar-shaped cells with a proteoglycan matrix located at the periphery, evenly distributed;<br>Score 1: partially stellar and partially round cells, more stellar than round;<br>Score 2: mostly large, round cells, separated by dense areas of proteoglycan matrix;<br>Score 3: large, round cells, separated by dense areas of proteoglycan matrix.
Morphology of the AF	Score 0: well-organized collagen lamellae with no ruptures;<br>Score 1: inward bulging, ruptured, or serpentine fibers constitute <25% of the af;<br>Score 2: inward bulging, ruptured, or serpentine fibers constitute 25−50% of the af;<br>Score 3: inward bulging, ruptured, or serpentine fibers constitute >50% of the af.
Cellularity of the AF	Score 0: fibroblasts comprise >90% of the cells;<br>Score 1: fibroblasts comprise >75–90% of the cells;<br>Score 2: intermediate;

	Score 3: chondrocytes comprise >75% of the cells.
Border between the NP and AF	Score 0: normal, without any interruption;<br>Score 1: minimal interruption;<br>Score 2: moderate interruption;<br>Score 3: severe interruption.

NP: Nucleus pulposus; AF: Annulus fibrosus
